# Nanozymes in the Treatment of Pediatric Inflammatory Diseases: Opportunities and Challenges

**DOI:** 10.3390/ph19071061

**Published:** 2026-07-09

**Authors:** Jiayan Zhang, Jing Zhou, Chuan Zhang, Bing Li, Chenghui Liu, Yuan Yong

**Affiliations:** 1 Nanomedicine Innovation Research and Transformation Institute, Affiliated Hospital of North Sichuan Medical College, Nanchong 637000, China; zhangjy20262026@163.com (J.Z.); zj8126118@yeah.net (J.Z.); zhangchuanforever@yeah.net (C.Z.); chongsuzhixin@163.com (B.L.); 2 Key Laboratory of Pollution Control Chemistry and Environmental Functional Materials for Qinghai-Tibet Plateau of National Ethnic Affairs Commission, School of Chemistry and Environment, Southwest Minzu University, Chengdu 610041, China

**Keywords:** nanomedicine, nanozymes, pediatric inflammatory diseases, antioxidation, integrated diagnosis and treatment

## Abstract

Pediatric inflammatory diseases, such as neonatal necrotizing enterocolitis (NEC), juvenile idiopathic arthritis (JIA), and asthma, pose numerous challenges in clinical treatment due to their complex pathogenesis and the still-developing stage of children’s immune systems. These challenges include poor precision, strong toxic and side effects, and unclear long-term biological safety. In recent years, the development of nanozymes has provided new opportunities to address these issues. This review systematically summarizes the latest research progress of nanozymes in the diagnosis and treatment of pediatric inflammatory diseases. Firstly, we focus on the rational design principles of nanozymes tailored to the physiological characteristics of children, including the regulation of their catalytic activity and strategies for optimizing biocompatibility. Subsequently, we deeply analyze the core roles of these antioxidant nanozymes in breaking the vicious cycle of oxidative stress and reshaping the inflammatory microenvironment through multiple mechanisms, such as scavenging reactive oxygen species (ROS), regulating macrophage polarization, and inhibiting the pro-inflammatory protein (NLRP3 inflammasome). Moreover, we detail the successful applications of nanozymes in various typical pediatric inflammatory disease models and emphasize their potential as a multifunctional theranostic platform in achieving synergistic antioxidant, antibacterial, and targeted drug delivery. Finally, this article prospectively discusses the key challenges that nanozymes must address in the process of clinical translation for children, including long-term biological safety, precise dose control, and individualized drug delivery, and looks forward to the development of intelligent responsive and biodegradable nanozymes. This review aims to provide theoretical basis and reference ideas for the in-depth exploration and clinical application of nanozymes as innovative nanomedicines in the field of pediatric precision medicine in the future.

## 1. Introduction

### 1.1. Clinical Status and Challenges of Inflammatory Diseases in Children

Inflammatory diseases in children belong to a group of disorders which are marked by abnormal or continuous inflammatory reactions caused by immune system imbalance. They encompass a wide range of conditions, from common acute infectious inflammations (such as pneumonia and tonsillitis) to chronic systemic inflammations (such as rheumatoid arthritis, atherosclerosis, and diabetes-related inflammation), as well as autoimmune diseases (such as systemic lupus erythematosus and psoriasis). In adults, these diseases are often closely related to factors such as aging, metabolic disorders, and environmental exposure, and their course is prolonged and prone to involve multiple organ systems. However, when inflammatory diseases occur in children, especially infants, their occurrence, development, and outcome show completely different physiological and pathological bases. Pediatric inflammatory diseases are a group of conditions resulting from an immature or dysregulated immune system, and they broadly include necrotizing enterocolitis (NEC) in newborns, juvenile idiopathic arthritis (JIA), asthma, and inflammatory bowel disease (IBD), among others. Compared with adults, children’s immune system is still in the stage of development and shaping, and its response pattern to inflammatory stimulation, tissue repair ability, and tolerance to therapeutic drugs are significantly different. This makes the diagnosis, treatment, and management of inflammatory diseases in children face more severe challenges, and constitutes a major problem in current pediatric clinical practice.

A thorough knowledge of the unique characteristics of the immune system in children is the basis for designing individualized treatment plans for inflammatory diseases in children. The immune system of children, especially in newborns and infants, is in a period of continuous development and maturation. On one hand, the functions of both innate and adaptive immunity have not been fully established, indicating immature immune cell functions and inadequate antibody production, which results in a reduced capacity to eliminate pathogens and a higher chance of infection as well as overreaction of inflammation [1]. On the other hand, the immune system has to be trained and build up immune tolerance when exposed to external antigens [2]. This “unsatisfactory” state causes children to be susceptible to immune imbalance, thus leading to different kinds of inflammatory and auto-immune diseases.

Additionally, conventional approaches to managing inflammatory diseases in children remain limited in clinical settings. Current regimens still depend heavily on broad-spectrum agents including glucocorticoids, non-steroidal anti-inflammatory drugs, and immunosuppressants. However, these drugs, while exerting therapeutic effects, also bring a series of bottlenecks: (1) Lack of specificity and severe side effects. Traditional drugs are distributed systemically and exert pharmacological effects in non-target tissues and organs, which may lead to growth inhibition, metabolic disorders, bone damage, and opportunistic infections in children, with long-term effects being particularly prominent in rapidly developing children [3]. (2) Simple extrapolation of “adult protocols”. Many drug dosages and regimens for children are based on extrapolation from adult data, without fully considering the unique pharmacokinetic and pharmacodynamic differences in children. This leads to uncertain efficacy or increased risk of side effects, significantly exacerbating the precision medicine dilemma of “same disease, different drugs; same drug, different dosages” in pediatrics. (3) Drug resistance and disease recurrence. For chronic diseases such as JIA and IBD, long-term medication can lead to drug resistance, and the recurrence rate is high after drug withdrawal. Therefore, there is an urgent need to develop new therapies that can intervene in the fundamental mechanisms of inflammatory diseases in children.

Currently, certain severe inflammatory diseases specific to children (such as NEC) pose a significant challenge to the existing treatment system. Take NEC as an example, which is more common in premature infants and is characterized by sudden onset, rapid disease progression, and extremely high mortality. The underlying pathology is driven by several interconnected factors. First, prematurity leaves the intestinal barrier structurally immature, creating an opportunity for bacterial translocation. This is compounded by dysbiosis, which disrupts local immune homeostasis. When superimposed with insults such as ischemia–reperfusion, these conditions trigger intense oxidative stress and uncontrolled inflammation. The interplay of these three elements ultimately results in transmural intestinal necrosis and can progress to multi-organ failure. However, current clinical treatments mainly rely on traditional methods (such as fasting and gastrointestinal decompression) and broad-spectrum antibiotics, which can only control secondary infections but are not adapted to effectively interrupt the core link in the vicious cycle, namely the “oxidative stress–inflammatory storm” axis. When medical treatment fails to stop the progressive necrosis of the intestinal tract, surgery becomes the last line of defense to save lives, but it is also accompanied by serious complications such as short bowel syndrome, postoperative growth retardation, and long-term neurodevelopmental disorders. Therefore, there is an urgent need in clinical practice to develop novel targeted therapeutic strategies that can actively intervene in the core pathways of oxidative stress and rapidly protect and repair the intestinal mucosal barrier at the early stage of the inflammatory cascade reaction, in order to fundamentally curb disease progression and improve the short-term and long-term prognosis of these critically ill children.

In summary, the clinical treatment of inflammatory diseases in children is facing multiple challenges: the particularity of the immune system, the limitations of traditional drugs, and the urgency of severe diseases. Therefore, breaking through the existing treatment bottlenecks of inflammatory diseases in children and collaboratively developing new diagnostic and therapeutic strategies that are highly efficient, precise, and safe has become a key issue that needs to be urgently addressed in this field. Against this backdrop, the emergence of nanozymes with enzyme-like catalytic activity provides an unprecedented opportunity to achieve the goal of efficient, precise, and safe treatment.

### 1.2. The Rise in Nanozymes: From Catalytic Materials to Biomedical Applications

**Figure 1:**
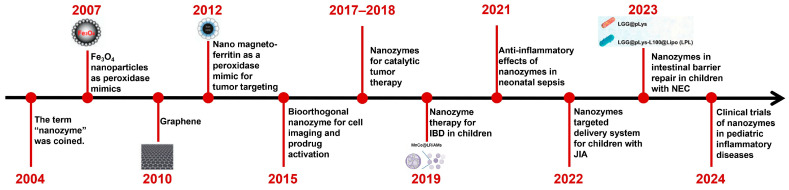
Key progress schedule of nanozymes in pediatric disease applications.

In 2007, Yan’s team discovered that magnetic iron oxide (Fe_3_O_4_) nanoparticles themselves possess catalytic activity similar to that of natural peroxidase [4]. This discovery broke the traditional notion that enzymes can only be proteins, announcing the birth of a new type of artificial mimetic enzyme–nanozymes. Over the next decade, many nanozymes based on inorganic, organic, and composite materials were proven to have the activities of various natural enzymes, and their catalytic mechanisms have been systematically studied by scientists. Their application scope has rapidly expanded from in vitro detection to complex biomedical fields. Compared with natural enzymes and small molecule drugs, nanozymes have a series of unique electrical structures and excellent physical and chemical properties. With the continuous development of nanozymes, an increasing number of them are gradually being used in the research of children’s diseases, thus becoming ideal candidates for the treatment of inflammatory diseases in children (Figure 1).

(1) Stability and low expense. Natural enzymes, being proteins, are affected by factors such as temperature and pH, and their preparation and purification processes are costly. In contrast, nanozymes exhibit exceptionally high stability and can withstand severe physiological conditions. As nanomaterials, they can be produced on a large scale under controlled conditions at low cost, and may find applications in medicine [5]. For instance, Du et al. constructed an “all-in-one” artificial enzyme by encapsulating Au/CeO_2_ nanorods within Cu-MOFs. This configuration significantly enhances the stability of the nanozymes in challenging environments, which is beneficial for maintaining their activity in complex biological media [6].

(2) Multifunctionality and Synergistic Effects. Natural enzymes usually have only a single catalytic function and play a highly specific role in a particular reaction system. In contrast, nanozymes, as a new type of nanomaterial, exhibit unique “multifunctional catalysis” due to their complex surface structure and diverse element composition. This characteristic is not only reflected in their ability to simulate the catalytic activities of various natural enzymes, such as peroxidase-like (POD), oxidase-like (OXD), and superoxide dismutase-like enzymes (SOD), but also enables flexible design and precise control of catalytic performance by adjusting their size, morphology, surface modification, and external environment. It is particularly worth discussing whether the binding mechanism between nanozymes and substrate is similar to that of natural enzymes or has essential differences. This question is crucial for understanding the catalytic nature of nanozymes. Natural enzymes rely on the hydrophobic pockets and hydrogen bond networks formed by their precisely folded three-dimensional structures to achieve specific recognition and binding to substrates through “lock-key” or induced fit mechanisms. In contrast, traditional nanozyme surfaces lack such clear substrate recognition sites and mainly rely on non-specific electrostatic adsorption or physical collision to activate substrates. Therefore, their substrate selectivity is usually much lower than that of natural enzymes, which is also one of the key bottlenecks restricting the practical application of nanozymes. In recent years, researchers have proposed the “modular structure” strategy to address this issue. That is, by separating and designing the catalytic active units from the substrate recognition units and flexibly assembling them, artificial substrate binding sites (ASBSs) can be constructed on the surface of nanozymes. Li et al. used molecular imprinting technology (MIT) and used levodopa as the template molecule to coat an imprinting polymer layer on the surface of Fe_3_O_4_ nanozymes, successfully preparing multifunctional molecularly imprinted nanozymes with ASBSs (MIPNMFs) [7]. This design mimics the “lock-key” relationship of natural enzymes: the cavities formed in the imprint layer are complementary in shape, size, and functional groups to the target substrate, enabling selective enrichment of substrate molecules near the active center of the nanoenzyme, demonstrating excellent substrate affinity and catalytic efficiency.

Moreover, the multifunctionality of nanozymes extends beyond catalysis, such as showing synergistic enhancement effects under external fields like light, heat, and electricity, enabling broad application prospects in bio-sensing, disease diagnosis and treatment, environmental management, and energy conversion. For instance, cerium dioxide nanozymes are a typical example, with their catalytic activity core lying in the reversible conversion between Ce^3+^ and Ce^4+^. In acidic inflammatory environments (such as synovial fluid of arthritis or ischemic tissues), the Ce^4+^ on the surface is more prone to be reduced to Ce^3+^, thereby preferentially exhibiting SOD activity, efficiently converting superoxide anion (·O_2_^−^) into hydrogen peroxide (H_2_O_2_). Furthermore, under neutral or alkaline conditions, the Ce^3+^ sites of it can catalyze the decomposition of H_2_O_2_ and show strong catalase (CAT) activity, changing it into harmless water and oxygen. This “switching” property allows it to fit the chemical surroundings of the inflammatory area and carry out a series of clearance for different reactive oxygen species (ROS), thus effectively breaking the oxidative stress vicious cycle [8]. In addition to intelligent response, some nanozymes are inherently capable of multiple synergistic activities. For example, trimanganese tetraoxide (Mn_3_O_4_) nanozymes have been reported to simulate the activities of three key antioxidant enzymes, namely SOD, CAT, and glutathione peroxidase (GPx). This means that Mn_3_O_4_ itself can independently complete the entire antioxidant chain reaction from eliminating initial ·O_2_^−^ to decomposing subsequent H_2_O_2_ and then reducing lipid peroxides, achieving multi-target, synergistic intervention of cellular oxidative stress, and thereby facilitating the reconstruction of redox homeostasis [9]. Moreover, nanozymes can utilize their excellent magnetic, optical properties, or drug loading capabilities to construct “diagnostic-therapeutic integration” or “multi-modal collaborative treatment” platforms. For example, manganese oxide (MnO_2_) nanozymes can undergo decomposition reactions in acidic and H_2_O_2_-rich inflammatory microenvironments. This process itself can consume inflammatory signaling molecules and produce Mn^2+^. On one hand, the generated Mn^2+^ is an excellent T1-weighted magnetic resonance imaging (MRI) contrast agent, making the inflammatory area significantly “brighter” on MRI images, enabling real-time, high-contrast visualization of the inflammatory site. On the other hand, its undecomposed MnO_2_ core continues to exert POD activity, alleviating tissue hypoxia and clearing ROS. Thus, through MRI, it is possible to directly monitor the distribution and therapeutic effect of the nanozyme in vivo, truly achieving “diagnostic-therapeutic integration” [10,11,12]. Additionally, researchers have developed a “three-in-one” nanoplatform that integrates chemotherapy, photothermal therapy, and catalytic therapy. On one hand, the hollow Prussian blue itself can simulate POD and CAT, performing catalytic therapy. Secondly, under near-infrared laser irradiation, it can convert light energy into heat energy, conducting local mild photothermal therapy on the inflammatory site, and also promoting the intelligent release of its anti-inflammatory drug dexamethasone, achieving a synergistic effect of catalysis, photothermal, and chemotherapy. In animal models of rheumatoid arthritis, its efficacy is far superior to any single therapy, achieving a synergistic therapeutic effect [13].

(3) Flexible catalytic ability. Because of the great heterogeneity of various disease microenvironments (for instance, the low acidity and high H_2_O_2_ content in the extracellular microenvironment of tumors while the low alkalinity and oxidative stress in neurodegenerative diseases), the conventional single-functional nanozymes usually do not work well. Hence, by precisely regulating the size, shape, surface chemistry, and element composition of the nanomaterials (like doping and alloying), the “designable” enzyme-like activity can be achieved, thus improving the catalytic efficiency, substrate selectivity, and specificity to meet the special requirements of different disease models [14]. For example, forming defects near the Fe-N_4_ sites can promote the charge transfer from iron atoms to the carbon matrix, making the iron center more active and facilitating its interaction with the reaction substrates (such as H_2_O_2_). Consequently, the reaction energy barrier can be significantly reduced and the catalytic rate enhanced [15]. At the same time, by incorporating the spontaneous polarization electric field of ferroelectric material BTO, a special local environment is established. This internal electric field can arrange and optimize the orientation of the reactant molecules, similar to the “electrostatic pre-organization effect” in the active sites of natural enzymes, thereby greatly speeding up the kinetic processes of enzymatic reactions [16].

(4) Easy surface functionalization. The chemical modification of the surface of nanozymes is one of the core methods for achieving functional customization and optimizing biomedical applications. It can be accomplished by connecting polyethylene glycol (PEG), targeting molecules (such as antibodies, peptide segments), or cell transmembrane peptides, thereby significantly improving their biocompatibility, prolonging their circulation time in the body, and enabling active targeted enrichment at specific diseased cells or tissues (such as activated macrophages, inflammatory endothelium). For example, Wu et al. carried out a covalent coupling of L-arginine and myricetin through the Mannich reaction, and then utilized non-covalent interactions to drive its self-assembly, thereby constructing carrier-free myricetin–arginine conjugated nanozymes (MANZs). These nanozymes can achieve selective targeting of M1-type macrophages through endocytosis mediated by the cationic amino acid transporter 2, and efficiently accumulate in inflammatory joint sites. At the same time, MANZs possess multimodal therapeutic effects for eliminating ROS, reversing the polarization of M1-type macrophages, and inhibiting osteoclast differentiation. In the collagen-induced arthritis (CIA) mouse model, MANZs can significantly alleviate joint swelling, synovitis, and bone erosion symptoms, and have no systemic toxicity. This work integrates cationic amino acids and natural polyphenols into a self-assembled target-specific nanoplatform with high biocompatibility and translational potential, laying a promising paradigm for the treatment of rheumatoid arthritis [17]. Additionally, another research team has constructed a docetaxel nanoliposomal carrier with folate and activated transmembrane peptide (ACPP) co-modification. Among them, ACPP can be specifically activated by matrix metalloproteinases MMP-2/9 secreted by tumor tissues, thereby achieving precise drug release at the tumor site [18].

In the treatment of pediatric inflammatory diseases, the production of oxidative stress not only directly damages the cells but also enhances the inflammatory signals in the diseased microenvironment. Fortunately, nanozymes can eliminate different kinds of ROS individually and effectively inhibit the generation of ROS chain reaction, thus protecting mainly the key biological macromolecules such as cell membranes, proteins, and DNA from oxidative damage, which provides important material assistance for maintaining the intracellular balance and preventing the vicious circle of inflammation. As shown in Table 1, according to the catalytic mechanism of these natural enzymes, researchers have produced various nanomaterials which mimic the enzymatic action and have confirmed their therapeutic effects in pediatric inflammatory models (for example, IBD, atopic dermatitis (AD), respiratory syncytial virus infection, etc.) [19,20]. These nanozymes, due to their four core advantages of stability, high catalytic efficiency, multi-functionality, and precise design, have paved the way for translating nanomaterial concepts into biomedical applications. Notably, applying them to pediatric inflammatory diseases holds promise for building intelligent platforms that actively modulate the inflammatory microenvironment, offering potential solutions to current clinical bottlenecks.

### 1.3. The Concept and Structure of the Review

**Figure 2:**
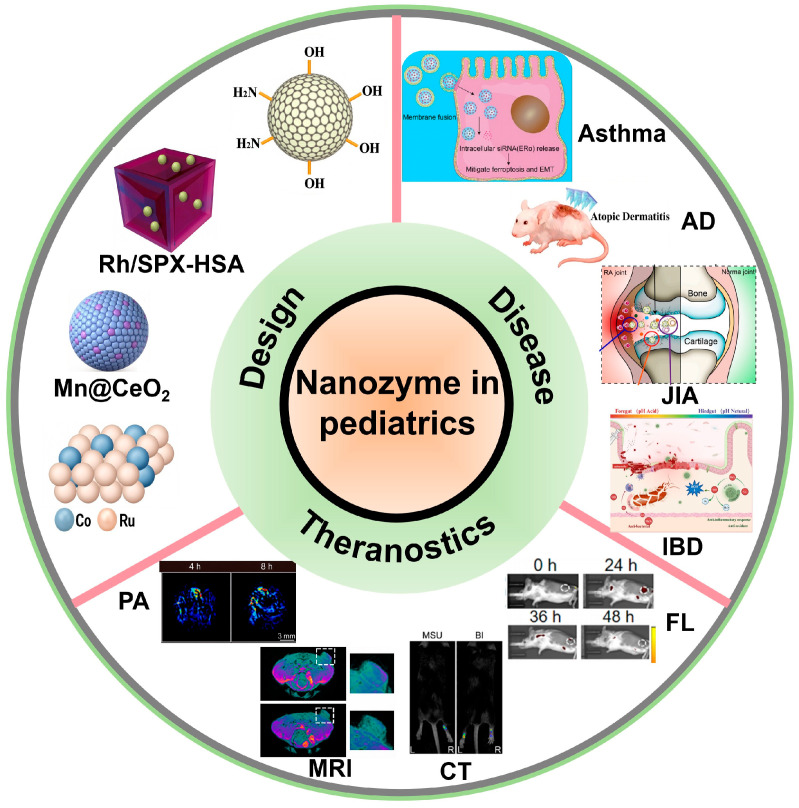
Schematic diagram of the review: From design, mechanism, and bioapplications to summarizing the development for nanozymes in pediatrics.

Although nanozymes have been widely applied in tumor catalytic therapy and antibacterial infections, their great potential in the specific field of pediatric inflammatory diseases has not been systematically explored and envisioned. The inherent uniqueness of the pediatric immune system and its corresponding disease spectrum determine that specific intervention strategies for inflammatory diseases must be completely different from those for adults. Currently, it is urgent to deeply connect the designability and multifunctionality of nanozymes with the clinical precise needs of pediatric diseases. Based on this, this review will for the first time systematically explore how to build an innovative diagnosis and treatment system for pediatric inflammatory diseases with nanozymes as the core, and strive to answer this core question from the theoretical framework to the potential for transformation. To clearly and deeply answer this question, this article will be discussed based on the following logical framework (Figure 2):

Firstly, we will elucidate the design principles and underlying mechanisms governing the use of nanozymes in treating pediatric inflammatory conditions. This chapter will not merely list the materials, but will focus on how to rationally design nanozymes with high catalytic efficiency, superior biological safety, and targeted properties based on the physiological characteristics of children and the microenvironment of specific diseases. The discussion will further address how these nanozymes achieve their anti-inflammatory effects at the molecular level—by eliminating ROS, directing immune cell differentiation, and interfering with crucial signaling cascades. Following this mechanistic overview, we will examine their emerging roles in specific pathological settings, highlighting state-of-the-art nanozyme strategies for treating distinct pediatric inflammatory conditions. From NEC to JIA, from asthma to IBD, this chapter will combine specific cases to empirically analyze the therapeutic efficacy and unique advantages of nanozymes in different pathological scenarios. Then, we will look forward to the frontier exploration and future development of nanozyme technology. We will focus on how it can overcome the single anti-inflammatory function and achieve integrated diagnosis and treatment through the integration of imaging probes or the loading of therapeutic drugs, thereby significantly improving the accuracy and therapeutic effect of pediatric disease management. Finally, we will critically assess the major hurdles and future prospects for translating nanozymes into pediatric clinical practice. Particular attention will be given to long-term biosafety, personalized dosing strategies, and ethical considerations. We will also offer forward-looking insights into emerging directions, such as stimuli-responsive and biodegradable nanozyme systems. Through this systematic review from the perspective of from simple to complex, from theory to practice, this overview aims to provide a comprehensive knowledge framework for researchers in nanomedicine, pediatrics, pharmacy, and materials science, stimulate new research ideas, and jointly promote the early application of nanozyme technology to benefit a large number of pediatric patients with inflammatory diseases.

### 1.4. A Brief Literature Retrieval and Analysis in This Field

This article aims to accurately identify the trend of nanozymes in the research of pediatric inflammatory diseases from both qualitative and quantitative perspectives. To achieve this, the following search terms were used to conduct a search in the Web of Science Core Collection: TS = (Nanomedicine OR nanozyme OR nanoparticles) and TS = (Pediatric inflammatory diseases OR antioxidation OR anti-inflammation OR integrated diagnosis and treatment). By leveraging the advanced search capabilities of Web of Science and its core collection database, the research direction was analyzed, and relevant keywords and typical inflammatory diseases for this study were selected. The retrieved literature is in the form of downloaded plain text files, and the records and citations are complete.

## 2. Design Strategies and Mechanism

**Figure 3:**
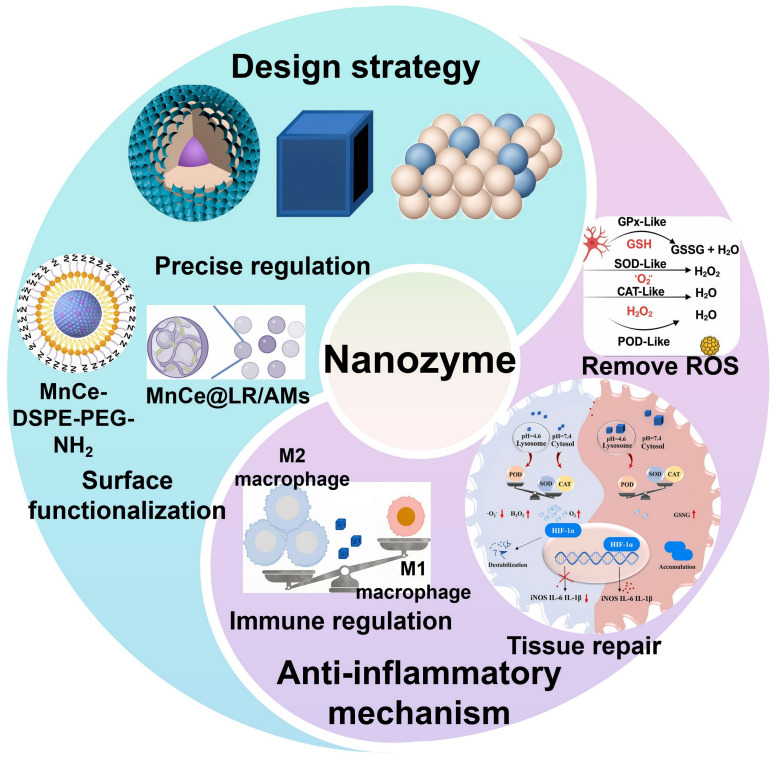
The main design strategy and anti-inflammatory mechanism of nanozymes.

The application of nanozymes in the treatment of inflammatory diseases in children has its core advantage in that it can achieve precise “top-down” design to meet the specific physiological and pathological characteristics and clinical needs of children, and customize nanozyme preparations with both high catalytic activity and high biological safety. This chapter will deeply explore the rational design strategies of nanozymes and systematically analyze their multi-level and multi-dimensional anti-inflammatory mechanism (Figure 3).

### 2.1. Rational Design Strategies for Intelligent Nanozymes

#### 2.1.1. Precise Regulation of the Core Catalytic Center

The catalytic active center of nanozymes is fundamental to their functional performance. Through material selection and engineering their microstructure, the activity can be precisely optimized. On one hand, material genomics selection and electronic structure regulation are carried out for different enzyme-like activities of different nanozymes. For example, noble metal (such as Pt, Au) nanozymes are renowned for their outstanding POD activity while metal oxides (such as CeO_2_, MnO_2_) often simulate SOD and CAT activities [36]. Meanwhile, the emerging single-atom enzymes (SAzymes) achieve nearly 100% atomic utilization and uniform active sites by dispersing the metal active center at the atomic level, significantly improving the catalytic efficiency. It is worth noting that the coordination environment and electronic structure of the surface atoms in single-atom nanozymes directly determine the strength of their SOD and CAT activities. Zhou et al. reported the Fe-based single-atom nanozyme (Fe-DMOF), with Fe-N_x_ as the active center, and the surface Fe atoms interact with the substrate molecules through axial coordination [21]. In the SOD pathway, the Fe site catalyzes the disproportionation of ·O_2_^−^ into H_2_O_2_ and O_2_. In the CAT pathway, the same Fe site decomposes H_2_O_2_ into H_2_O and O_2_ through a two-step proton-coupled electron transfer process. Furthermore, Xiao et al. constructed a nanozyme (RuSA + AC) on ultrathin two-dimensional g-C_3_N_4_ with both Ru-N_5_ monometallic sites and Ru atomic clusters using the “ligand-mediated polymerization limitation” strategy. Density functional theory (DFT) calculations indicated that the Ru-N_5_ structure with axial N coordination optimized the d-band center position of Ru atoms, enhanced the overlap of Ru 4d orbitals and oxygen intermediate p orbitals, thereby reducing the energy barriers of the key steps in SOD and CAT catalytic reactions [22]. This study revealed the fine regulatory role of surface atomic coordination numbers and axial ligand types on enzyme activity. Appropriate coordination strength can both stabilize monometallic active sites and optimize the kinetic balance between substrate adsorption and product desorption. It is particularly noteworthy that the synergistic operation mechanism of the activities of SOD and CAT enzymes is the key to the efficient removal of ROS by nanozymes. Specifically, the activity of SOD first catalyzes the disproportionation reaction of ·O_2_^−^, converting it into hydrogen peroxide H_2_O_2_ and O_2_: 2 ·O_2_^−^ + 2 H^+^ → H_2_O_2_ + O_2_. However, the generated H_2_O_2_ itself is also a highly oxidizing ROS. If it is not promptly removed, it will still cause damage to the cells. At this time, the CAT activity on the nanozymes then takes over and rapidly decomposes H_2_O_2_ into harmless H_2_O and O_2_: 2 H_2_O_2_ → 2 H_2_O + O_2_. This complete cascade reaction (SOD to CAT) ensures the complete detoxification from the highly toxic ·O_2_^−^ to the final product, avoiding the accumulation of the intermediate product H_2_O_2_.

For instance, the abundant Ce^3+^/Ce^4+^ valence state conversion on the surface of CeO_2_ nanozymes precisely enables the simultaneous support of these two reactions: the Ce^3+^ sites tend to catalyze the SOD reaction, while the Ce^4+^ sites are conducive to the progress of the CAT reaction. This spatially adjacent and chemically complementary dual-functional active center enables the CeO_2_ nanozymes to achieve an efficient ROS metabolic cycle of “one-step generation and one-step elimination” on the same particle. For example, the Mn@CeO_2_ nanozyme constructed by doping manganese (Mn) atoms in the CeO_2_ lattice not only retains the inherent SOD/CAT activity of cerium oxide, but also the lattice distortion and electron interaction introduced by doping further optimize its ability to eliminate ROS [23]. At the same time, size, morphology, and crystal plane effects strongly influence the catalytic performance of nanozymes. Smaller nanozymes, such as the 3 nm Prussian blue nanozymes (PBNZs) reported here, tend to accumulate intracellularly and often display enhanced catalytic efficiency owing to their higher specific surface area [24]. In addition, exposing specific crystal facets provides an effective means to tune both catalytic activity and selectivity. For example, the different crystal planes of ruthenium–cobalt dual-metal nanosheets (RuCo NSs), respectively, dominate the CAT and SOD-CAT cascade activities, achieving precise division of the antioxidant pathway [25].

#### 2.1.2. Surface Engineering and Intelligent Response Design

For pediatric applications, improving the biocompatibility and targeting ability of nanozymes while minimizing their non-specific toxicity is especially critical. This is not only a key requirement for moving nanozymes from basic research into pediatric clinical practice, but also stems from children’s unique physiological traits and health needs. One approach is to coat the nanozyme surface with PEG or other biocompatible polymers, like sodium alginate. This gives nanozymes a kind of “stealth” property, helping them circulate longer in the body and reducing immune recognition and clearance. For example, a manganese-doped ceria (Mn@CeO_2_) nanozyme–probiotic system encapsulated in alginate microspheres can effectively shield probiotics from stomach acid damage. At the same time, the negative charge of alginate and its ability to bind to receptors at inflamed sites allow dual-targeted delivery to intestinal inflammation [37]. However, merely enhancing biocompatibility and targeting is not sufficient to meet the stringent requirements of pediatric precision treatment. The innovative design of nanozymes must deeply explore the process of surface modification of nanozymes by selecting appropriate enzymes to further enhance their catalytic activity. A pioneering study recently published in *Advanced Materials* provides an extremely inspiring example: Mehta et al. successfully constructed a new type of “super nanozyme” (CeO_2_ NPs-GOx) by covalently coupling glucose oxidase (GOx) to the surface of cerium oxide nanoparticles (CeO_2_ NPs) [26]. The brilliance of this design lies in its ingenious utilization of the cascade catalytic reaction between GOx and CeO_2_ NPs. Specifically, GOx first catalyzes the oxidation of glucose in the wound environment, generating H_2_O_2_ and gluconic acid; subsequently, CeO_2_ NPs utilize the H_2_O_2_ produced by GOx and the O_2_ generated from its self-decomposition, with the assistance of ATP, to continuously produce a large amount of ·O_2_^−^ and ·OH. This “one-two punch” strategy not only overcomes the bottleneck of low catalytic activity of traditional cerium oxide nanozymes at neutral physiological pH, but also, through the introduction of GOx, achieves a specific response to the glucose substrate, thereby precisely amplifying the production of ROS at the infection site. This case profoundly reveals the paradigm shift in the surface modification strategy: from merely pursuing “invisibility” and “targeting”, it has shifted to leveraging the catalytic activity of biological enzymes to empower nanozymes and construct a new type of “intelligent response-cascade amplification” diagnostic and therapeutic platform. Inspired by this, future nanozyme design for children can further explore coupling more types of functional enzymes (such as SOD, CAT, hyaluronidase, etc.) with nanozymes to achieve more complex multi-enzyme cascade reactions, thereby more precisely regulating the pathological microenvironment. For more precise treatment, targeting molecules such as antibodies or peptide fragments can be attached to the nanozyme surface, enabling active accumulation at inflammatory sites. An even more advanced design involves creating environment-responsive “smart switches” keeping nanozymes inactive until they reach the target area, where they are activated by specific pathological signals (such as low pH, high ROS levels, or particular enzymes present in inflamed tissue). This strategy greatly reduces off-target effects on healthy tissues, which is especially important for young patients whose organs are still in key developmental stages.

#### 2.1.3. Types and Applications of Nanozymes

Nanozymes, with their diverse material compositions and adjustable catalytic activities, have demonstrated multi-dimensional application potential in the diagnosis and treatment of inflammatory diseases in children. According to the type of material, nanozymes can be divided into metal-based nanozymes (such as Pt, Au, Pd, and other noble metal nanozymes), metal oxide nanozymes (such as Fe_3_O_4_, CeO_2_, MnO_2_, Mn_3_O_4_), carbon-based nanozymes (such as graphene quantum dots, carbon dots, and carbon nanotubes), metal–organic framework (MOF) -derived nanozymes, narrow-band semiconductor nanozymes (such as MoS_2_, WS_2_ and BiOI), and composite nanozymes (such as core–shell structure, heterojunctions, and nanozyme–polymer hybrid), as well as emerging single-atom nanozymes such as Fe-N_4_, Ru-N_5_, Cu-N_4_, etc. Different types of nanozymes have unique applications in antioxidant, anti-inflammatory, antibacterial, and diagnostic applications due to their unique electronic structure, coordination environment, and surface chemistry.

As antioxidants, nanozymes protect children’s developing tissues from oxidative damage by scavenging excessive ROS produced in the inflammatory microenvironment by mimicking SOD and CAT activity. With the reversible valence conversion of Ce^3+^/Ce^4+^, CeO_2_ can convert highly toxic ·O_2_^−^ to H_2_O and O_2_ via a SOD-to-CAT cascade in the same particle. Single-atom nanozymes achieve close to 100% atom utilization through atomically dispersed active centers, and the coordination environment of surface atoms directly determines the strength of SOD and CAT activities. For example, the Ru-N_5_ monatomic site optimized the d-band center position through the axial N coordination, reducing the SOD rate-limiting step energy barrier to 0.22 eV and CAT rate-limiting step energy barrier to 0.32 eV, significantly exceeding the catalytic efficiency of conventional metal oxide nanozymes [22]. Narrowband semiconductor nanozymes, such as MoS_2_ nanosheets, can simulate SOD, CAT, and POD activities simultaneously due to their abundant edge active sites and broad spectral absorption ability, further amplifying the ROS scavenging efficiency under visible light excitation [38].

As anti-inflammatory agents, nanozymes drive the transformation of macrophages from a pro-inflammatory (M1) phenotype to an anti-inflammatory (M2) phenotype and regulate the balance of T cell subsets by scavenging ROS and regulating immune cell signaling pathways. MnO_2_ nanozyme treatment reduced the secretion of TNF-α by about 74% and increased the secretion of IL-10 by about 4.8-fold in LPS-stimulated macrophages. Composite nanozymes, such as lamina-modified Pt nanozymes (Pt@LA), scour ROS through the SOD/CAT activity of the platinum core, and inhibit the Syk/NF-κB inflammatory signaling pathway by targeting the Dectin-1 receptor on the surface of microglia using laminae polysaccharide, thereby blocking the polarization of microglia to the pro-inflammatory M1 phenotype. Narrow-band semiconductor nanozymes, such as BiOI nanosheets, can produce local thermal effects under near-infrared light, synergize with their intrinsic POD-like activity to inhibit nuclear translocation of NF-κB and expression of pro-inflammatory factors, showing the advantages of photothermia-catalysis synergistic anti-inflammatory [27].

As antibacterial agents, nanozymes catalyze H_2_O_2_ to produce highly toxic ·OH through POD activity, directly damaging bacterial cell membranes and biofilms, and synergistic with traditional antibiotics. The minimal inhibitory concentration (MIC) of Fe_3_O_4_ nanozymes combined with vancomycin decreased from 2 μg/mL to 0.25 μg/mL (an 8-fold reduction), and the combined inhibitory index (FICI) was 0.375, which confirmed the synergistic effect. Due to their high density of Fe active centers and three-dimensional porous structure, MOF-derived nanozymes (Fe-MOF) exhibited stronger POD-like activity (4.2-fold increase in ·OH yield) than conventional Fe_3_O_4_ in the weak acidic bacterial microenvironment and effectively degraded S. aureus biofilms (81% reduction in biofilm biomass).

For diagnostic purposes, nanozymes can achieve highly sensitive detection of inflammatory biomarkers by virtue of their inherent physicochemical properties. MnO_2_ nanozymes decompose in the acidic H_2_O_2_ environment to produce Mn^2+^, which can be used as a T1-weighted magnetic resonance imaging (MRI) contrast agent to monitor the distribution of nanozymes in inflammatory sites and the therapeutic effect in real time. PBNZs can be used for photoacoustic imaging due to their strong near-infrared absorption, while carbon dot nanozymes can reflect the ROS level through the change in fluorescence signal. Narrow-band semiconductor nanozymes (such as MoS_2_ quantum dots) have both fluorescence emission and enzyme-like activity, which can realize the integration of “detect-treatment” diagnosis and treatment platform. Single-atom nanozymes (such as Fe-N_4_/C) have been used to construct highly sensitive electrochemical sensors for the trace detection of inflammatory markers (e.g., H_2_O_2_, NO, glucose) down to the nanomolar level due to their homogeneous active site and excellent electrocatalytic activity. These diagnostic functions provide strong technical support for early screening, accurate evaluation, and treatment monitoring of inflammatory diseases in children.

### 2.2. The Main Anti-Inflammatory Mechanism

The anti-inflammatory effect of nanozymes extends well beyond simple “antioxidant” activity. They can actively interfere with inflammatory signaling pathways, influence immune cell behavior, and reshape the inflamed environment on multiple levels. This is especially relevant for childhood conditions such as pediatric IBD, asthma, AD, and post-infection inflammatory storms. In these cases, nanozymes help reduce tissue damage by clearing excess ROS and curbing the release of pro-inflammatory factors. At the same time, they avoid the systemic side effects that often accompany long-term use of traditional steroids or immunosuppressants. Thus, nanozymes represent a safer and more precise treatment option for young patients.

#### 2.2.1. Elimination of ROS and Breaking the Malignant Cycle of Oxidative Stress

Oxidative stress is the common pathological basis of most inflammatory diseases. The core anti-inflammatory mechanism of nanozymes lies in their powerful and customizable antioxidant capabilities. On one hand, many nanozymes can simulate the natural antioxidant enzyme cascade in the human body. For example, Mn@CeO_2_ nanozymes and RuCo NSs [23,25] can successively simulate the activities of SOD and CAT. First, they catalyze the disproportionation of highly aggressive ·O_2_^−^ into H_2_O_2_, and then decompose H_2_O_2_ into harmless water and oxygen. This cascade reaction achieves a thorough removal of ROS, fundamentally breaking the vicious cycle of oxidative stress and avoiding the deficiency of small molecule antioxidants in “treating the symptoms but not the root cause”. Moreover, some nanozyme designs can even simultaneously remove ROS and reactive nitrogen species (RNSs), demonstrating a broad-spectrum antioxidant effect, providing the possibility for controlling complex inflammatory networks. This broad-spectrum antioxidant ability makes it have great potential in treating pediatric inflammatory diseases accompanied by complex nitrosative oxidative stress (such as sepsis, acute lung injury, neuroinflammatory diseases, etc.).

#### 2.2.2. Regulating the Function of Immune Cells

In pediatric inflammatory diseases, the primary issue is often an overactive or dysfunctional immune system. Nanozymes can directly interact with immune cells and help restore a healthy immune environment. Macrophages, in particular, play a key coordinating role in inflammation. By clearing ROS and modulating cell signaling pathways, nanozymes can encourage macrophages to shift from a pro-inflammatory (M1) state to an anti-inflammatory (M2) state. This helps break the vicious cycle of inflammation and restore immune balance.

Firstly, inflammatory diseases in children exhibit unique immune pathological characteristics, with macrophages playing a crucial role as a “bidirectional switch”. In diseases such as JIA, IBD, and NEC, macrophages not only serve as the main source of inflammatory factors (such as TNF-α, IL-6, IL-1β), but also act as a bridge connecting innate immunity and adaptive immunity. When macrophages are over-activated to the M1 phenotype, they produce large amounts of ROS and pro-inflammatory factors, leading to tissue damage and continuous amplification of inflammation. For children in the growth and development stage, this persistent immune imbalance not only damages local tissues (such as intestinal mucosa, joint synovium), but also interferes with the development of the entire immune system. For example, differently sized PBNZs exhibit differentiated enzyme-like activities due to their different distribution positions within cells, thereby specifically influencing the polarization direction of macrophages [24]. Small-sized PBNZs (~3 nm) can promote the transformation of macrophages from the pro-inflammatory M1 type to the anti-inflammatory M2 type, thereby actively inhibiting inflammation and initiating the tissue repair process. From a quantitative point of view, the regulatory effect of nanozymes on macrophage polarization can be quantitatively evaluated by a number of indicators. In the case of CeO_2_ nanozymes, treatment of LPS-stimulated RAW264.7 macrophages with CeO_2_ nanozymes at a concentration of 10 μg/mL for 24 h reduced the intracellular ROS level to about 35% of that of the control group, and decreased the mRNA expression of M1 marker iNOS by about 3.8-fold (detected by qPCR). The mRNA expression of Arg-1, a marker of M2, increased about 5.2-fold. The proportion of CD206-positive cells increased from 12.3% to 58.7%, while the proportion of CD86-positive cells decreased from 67.8% to 19.4% (detected by flow cytometry). At the molecular level, nanozymes regulate multiple signaling pathways to achieve macrophage polarization. Among them, the NF-κB signaling pathway is one of the most critical targets: IκBα protein was phosphorylated and degraded within 30 min after LPS stimulation, while CeO_2_ nanoenzyme pretreatment restored IκBα protein level to 82% of the basal level after 60 min, thereby inhibiting the nuclear translocation of p65 subunit. At the same time, the nanozymes promoted M2 polarization by activating the Nrf2/HO-1 antioxidant pathway: the nuclear Nrf2 protein content in the treated group peaked at 4 h after treatment (about 4.3-fold of the control), and the protein expression of HO-1 increased about 3.1-fold. In addition, the phosphorylation levels of STAT3 and STAT6 were also significantly upregulated (p-STAT3/STAT3 ratio increased by about 2.8-fold, p-STAT6/STAT6 ratio increased by about 3.4-fold), which further drove the transcriptional activation of M2-related genes (such as IL-10, TGF-β, Fizz1).

Beyond macrophages, nanozymes can also keep neutrophils from becoming excessively active and from releasing neutrophil extracellular traps (NETs) by clearing ROS and through other mechanisms. At the same time, they help balance different T cell subsets (such as Th1, Th2, Th17) and Treg cells, restoring immune balance from multiple angles. Take acute lung injury as an example. In one study, researchers injected CeO_2_ nanozymes intravenously and found that they accumulated effectively in the inflamed lungs. Thanks to their SOD and CAT activities, the nanozymes greatly lowered ROS levels in the lung tissue. This not only reduced damage to endothelial cells but also directly restrained excessive neutrophil activation and NET release. As a result, lung edema and inflammatory cell infiltration were significantly relieved [28,39]. At the quantitative level, CeO_2_ nanozymes treatment reduced the neutrophil count in bronchoalveolar lavage fluid (BALF) by about 79%, MPO activity by about 74%, and the level of NETs marker (cfDNA/MPO complex) by about 75%. Mechanologically, the nanozyme directly inhibited the activation of the PKCδ/NADPH oxidase pathway in neutrophils by eliminating ROS—the protein level of p-PKCδ decreased by approximately 70% and the expression level of NOX2 decreased by approximately 55%, thereby blocking the upstream signal for NETs formation. Moreover, there is also evidence from allergic asthma research, a disease driven mainly by a Th2-type immune response. In one study, researchers used MnO_2_ nanozymes loaded with dexamethasone. MnO_2_ breaks down in acidic and high-H_2_O_2_ conditions, which not only consumes H_2_O_2_ but also produces oxygen to help relieve tissue hypoxia. As a result, levels of Th2-type cytokines (such as IL-4, IL-5, and IL-13) in the lungs dropped significantly, while the expansion and function of Treg cells were boosted. This led to a clear improvement in disease symptoms. The study showed that nanozymes can directly correct the Th2/Treg imbalance by modulating oxidative stress and the local microenvironment, thereby easing airway inflammation [29]. Quantitative data showed that the concentration of IL-4, IL-5, and IL-13 in BALF decreased by 77%, 76%, and 77%, respectively, while the percentage of Treg cells (CD4^+^Foxp3^+^) in lung increased from 4.1% to 12.8% (a 3.1-fold increase). Foxp3 mRNA expression increased about 4.6 times. Mechanistically, MnO_2_ nanozymes suppressed the expression of OX40L in dendritic cells (DCs) by reducing the local H_2_O_2_ concentration (flow cytometry showed that the proportion of OX40L positive DCs decreased from 48.3% to 16.7%), thereby attenuating the promotion of Th2 differentiation by DCs. At the same time, it promoted the expansion of Treg cells by relieving the inhibition of Treg differentiation by oxidative stress (Nrf2 activation increased the histone acetylation level of FOXP3 gene promoter region by about 2.3-fold). In experimental autoimmune encephalomyelitis (EAE, an animal model of multiple sclerosis), PBNZs were found to clear H_2_O_2_ through their CAT activity, and were discovered to be able to inhibit the pathogenicity of myelin-specific Th1 and Th17 cells, while promoting the expansion and function of Treg cells, thereby significantly improving disease symptoms. This indicates that nanozymes also have therapeutic potential in Th1/Th17-driven autoimmune diseases.

#### 2.2.3. Synergistic Mechanism by Repairing Organ Barriers and Restoring Microbial Ecology

For pediatric-specific diseases, such as NEC and IBD, protecting the gut barrier and keeping the microbiota balanced is extremely important. In one study on IBD, Wang and colleagues found that a delivery system combining Mn@CeO_2_ nanozymes with probiotics (MnCe@LR/AMs) helped boost the expression of tight junction proteins such as Occludin and ZO-1. This repaired the damaged intestinal epithelial barrier and reduced the leak of harmful substances into the gut cavity. Furthermore, this system also helped create a healthier intestinal environment, as it changed the composition of the gut microbiota, increasing the numbers of beneficial bacteria like Bifidobacterium and Clostridium, while lowering the proportion of pro-inflammatory bacteria such as Escherichia coli. By using this “antibacterial plus probiotic” strategy, the researchers offered a new way to tackle inflammation right at its source [23]. It is worth noting that while the above nanozymes catalyze the removal of ROS, the large number of pro-inflammatory cytokines (such as IL-6, IL-8, HMGB1) in the inflammatory microenvironment are also key factors driving intestinal barrier damage and immune dysregulation. In recent years, heparin and its derivatives have been reported to be effective anti-inflammatory agents. One of the core mechanisms of heparin is that it can directly bind and neutralize a variety of pro-inflammatory cytokines through its highly sulfated negatively charged structure, thereby blocking their interaction with the corresponding receptors and the activation of downstream signaling pathways [40]. For example, heparin can interfere with the formation of the IL-6/IL-6Rα/gp130 signaling complex by binding to IL-6 and IL-6 receptor α subunit (IL-6Rα), thereby inhibiting the excessive activation of the JAK/STAT3 pathway and reducing the expression of pro-inflammatory genes. Similarly, the binding of heparin to IL-8 hinders its interaction with the CXCR2 receptor and impairs neutrophil recruitment and activation, thereby mitigating tissue damage. In addition, heparin can also bind to high mobility group protein B1 (HMGB1) and inhibit its secretion from macrophages and its binding to cell surface receptors, thereby blocking the amplification effect of HMGB1-mediated inflammation. Based on the above mechanism, the integration of heparin or its derivatives with nanozymes to construct a synergistic therapeutic platform is expected to realize the dual anti-inflammatory strategy of “catalytic removal of ROS + cytokine neutralization”. On the one hand, nanozymes (such as Mn@CeO_2_) efficiently remove ROS from inflammatory sites through their SOD-like and CAT-like activities, breaking the vicious cycle of oxidative stress. On the other hand, heparin or its derivatives inhibit the transmission and amplification of inflammatory signals at the source by binding and neutralizing key pro-inflammatory factors such as IL-6, IL-8, and HMGB1. This synergistic effect not only can more comprehensively regulate the inflammatory microenvironment, eliminate oxidative damage, and contain cytokine storm, but is also expected to play a synergistic role of “1 + 1 > 2” in protecting the intestinal barrier and regulating immune imbalance, providing a more comprehensive and precise new strategy for the treatment of inflammatory diseases such as NEC and IBD in children.

In conclusion, nanozymes offer a highly flexible “toolbox” for treating inflammatory diseases in children (Figure 3). By carefully designing their catalytic core and surface properties, we can create nano-drugs that are tailored to respond intelligently to the unique pathological environment of pediatric patients. These well-designed nanozymes work through a combination of mechanisms such as scavenging ROS, regulating immune responses, repairing barriers, and interfering with signaling pathways to tackle inflammation in a comprehensive and deep-rooted manner. This treatment approach goes beyond traditional single-target drugs and holds the promise of bringing transformative change to pediatric clinical practice.

## 3. The Application of Nanozymes in Specific Inflammatory Diseases of Children

The ultimate value of theoretical design and mechanistic studies can only be tested and applied in actual disease models. This chapter will focus on several typical pediatric inflammatory diseases where clinical needs are urgent. We will show how nanozymes, with their unique catalytic properties, can offer new solutions to these specific challenges. We strive to reveal the logical chain from “nanozyme design” to “in vivo efficacy” through case studies.

### 3.1. Autism Spectrum Disorder (ASD)

ASD is a common neurodevelopmental condition in children, with underlying causes closely linked to oxidative stress, brain inflammation, and neuronal cell death. However, adult mice (such as BTBR or VPA-induced models) have been used in the preclinical studies of nanozymes intervention in ASD, and there is no pediatric evidence that is not directly obtained from clinical young/neonatal animals or pediatric patients. The current conclusion of nanozyme anti-ASD is extrapolated from adult ASD animal models to the pediatric population. Age differences in children’s blood–brain barrier development, brain redox baseline, and immune maturity should be carefully considered in interpretation. Chen and colleagues developed a nanocatalyst composed of calcium hexacyanoferrate (CaH NCs). These nanocatalysts can mimic four major antioxidant enzymes: SOD, POD, CAT, and GPx, thereby creating a powerful chain reaction of antioxidant activity [30]. The proposed mechanism is as follows: CaH NCs remove excessive ROS, regulate mitochondrial membrane potential, upregulate the anti-apoptotic protein Bcl-2, and inhibit pro-apoptotic proteins such as caspase-3, effectively reducing neuronal apoptosis. In addition, these nanocatalysts modulate immune responses by increasing levels of the anti-inflammatory cytokine IL-10 and decreasing pro-inflammatory cytokines. They also curb the overactivation of microglia and astrocytes, thereby alleviating neuroinflammation. Consequently, ASD model animals exhibited significant improvements in social impairments, anxiety-related behaviors, and repetitive stereotyped movements. Although it can improve social dysfunction, anxiety, and repetitive behaviors in adult ASD mouse models, this is indirect evidence from adult animal models. Furthermore, its efficacy and safety in the developing brain of children still need to be verified in young animal models or pediatric clinical studies. In summary, targeting the oxidative stress–neuroinflammation axis with nanozymes provides a proof-of-concept intervention strategy for ASD, a common neurodevelopmental disorder in children. In the future, it is urgent to establish developmental/young animal ASD models and conduct pediatric safety and pharmacokinetics evaluation to fill the gap in the evidence chain of true pediatrics.

### 3.2. Intracerebral Hemorrhage (ICH)

Intracerebral hemorrhage (ICH) is an acute and devastating cerebrovascular disease, in which sudden blood aggregation in the pathological process can trigger a strong neuroinflammatory cascade. Nanozymes have shown unique therapeutic potential in this field. It is worth noting that all the current preclinical evidence for the efficacy of nanozymes intervention in ICH comes from adult rat or mouse collagenase/autologous blood injection models, and there are no pediatric-specific data obtained in young animal or pediatric ICH models. Therefore, the existing conclusion of nanozymes anti-ICH is extrapolated from the experimental results of adult animals to the children, and the interpretation should take into account the differences in the development of the blood–brain barrier in children, the baseline of brain oxidative stress, and the age specificity of the time window of secondary brain injury [31]. The beauty of the design lies in its dual mechanism of action: On the one hand, due to the inherent SOD-like and CAT-like activities of platinum core, Pt@LA can efficiently remove the excessive ROS including ·O_2_^−^, H_2_O_2_, and ·OH produced in the ICH microenvironment, with a SOD-like specific activity of 115 U/mg and a CAT-like specific activity of 3.11 U/mg, effectively alleviating oxidative stress damage to brain tissue. On the other hand, as a natural antagonist of Dectin-1 receptor, laminarin can specifically bind to the highly expressed Dectin-1 receptor on the surface of microglia and inhibit the activation of the downstream Syk/NF-κB inflammatory signaling pathway, thereby blocking the polarization of microglia to the pro-inflammatory M1 phenotype. Transcriptome analysis further revealed that Pt@LA exerted its neuroprotective effect by regulating the expression of immune-related genes and the neuroactive ligand–receptor interaction pathway. This study provides important implications for the application of nanozymes in children with intracerebral hemorrhage and related neuroinflammatory diseases. All the above mechanisms and efficacy have been verified in the adult rat ICH model, which belongs to the extrapolation evidence from the adult/adult animal system, and has not been replicated in the pediatric or juvenile ICH model. Although intracerebral hemorrhage in children is less common than that in adults, its etiology is complex (such as vascular malformation, coagulopathy, trauma, etc.). The children’s nervous system is in a critical period of development, which is less tolerant to oxidative stress and neuroinflammation, and the secondary brain injury is often more serious. Traditional antioxidant and anti-inflammatory therapies have limited efficacy in children with intracerebral hemorrhage, mainly due to the existence of the blood–brain barrier that limits the effective delivery of drugs and the lack of precise intervention methods for children’s unique pathophysiological characteristics.

### 3.3. Allergic and Respiratory Inflammatory Diseases

Childhood asthma, a chronic airway inflammation, is associated with Th2 immune responses, oxidative stress, and airway epithelial remodeling. In their study, Xie et al. discovered that airway epithelial cells from asthma patients exhibit a marked increase in estrogen receptor α (ERα) expression. This elevation in ERα activates the ferroptosis-to-epithelial–mesenchymal transition pathway, thereby exacerbating airway inflammation and remodeling. Furthermore, the team developed a cell membrane-mimicking nano-targeted delivery system that precisely delivers small interfering RNA (siRNA) to airway epithelial cells via “homo-targeting” to knock down the ERα gene. This approach successfully blocked the ferroptosis pathway and prevented epithelial–mesenchymal transition in the airways. Animal models confirmed that this treatment is both effective and safe for asthma [41]. Although childhood asthma has a higher prevalence, more prominent oxidative stress characteristics, and the risk of airway remodeling, there is no specific validation of nanozymes or nano-drug carriers in young animal models of childhood asthma. In the future, it is urgent to establish a developmental/young mouse model of allergic asthma and evaluate the age-stratified safety and lung targeting efficiency of nano-formulations to fill the gap in the evidence chain of nanomedicine in true pediatric asthma.

### 3.4. Atopic Dermatitis (AD)

AD is a chronic, relapsing inflammatory skin condition involving a combination of skin barrier dysfunction, a Th2-skewed immune response, and oxidative stress. These three factors feed into one another, creating a complex network that drives the onset, progression, and persistence of the disease. It is worth noting that the current preclinical evidence for the efficacy of nanozymes in the treatment of AD is derived from adult mouse models (induced by DNCB or MC903), and there is no pediatric-specific efficacy data of nanozymes in young animals or pediatric AD patients. The existing conclusions about the anti-AD efficacy of nanozymes in adult animals are extrapolated to the pediatric population. Age differences in epidermal thickness, transepidermal water loss rate, skin immune composition, and barrier maturity should be considered in the interpretation of children’s skin. To address this, researchers developed near-infrared (NIR) responsive microneedles (MNs) loaded with polydopamine (PDA) nanozymes. These microneedles can painlessly cross the skin’s stratum corneum and deliver the nanozymes directly to the affected area. Once there, the PDA nanozymes utilize their catechol and imino groups to efficiently scavenge various ROS (e.g., H_2_O_2_, ·O_2_^−^, and ·OH), thereby reducing oxidative stress. In addition, under NIR irradiation, the PDA nanozymes produce a photothermal effect. This not only helps suppress bacterial growth and alleviate inflammation but also improves local blood circulation and enhances the overall treatment outcome [32]. In the future, it is urgently necessary to establish a developmental/young mouse MC903 AD model and evaluate the age-stratified safety and efficacy of transdermal delivery of nanozymes, in order to fill the gap in the true pediatric AD evidence chain of nanozymes.

### 3.5. Joint Inflammatory Diseases (JIA)

**Figure 4:**
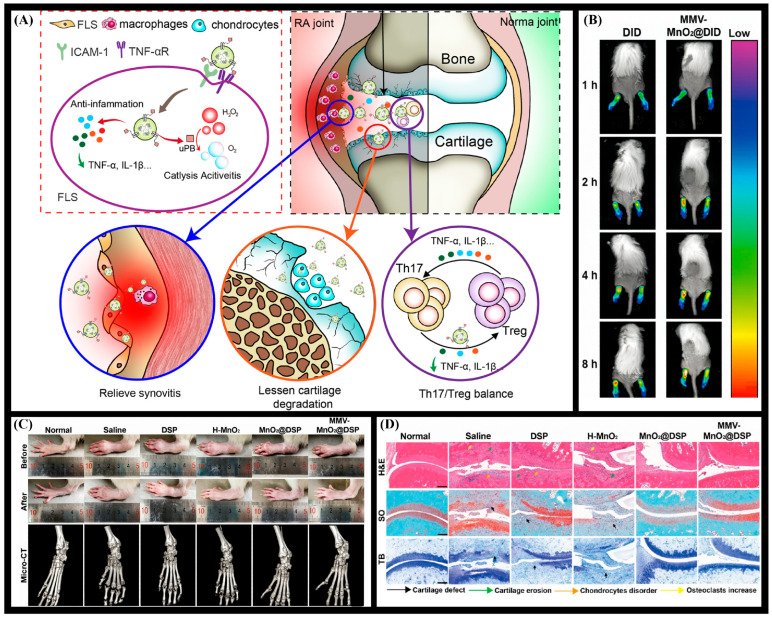
(**A**) Schematic diagram of nanozymatically engineered neutrophil-derived exosomes modulating the inflammatory environment to alleviate joint damage in advanced rheumatoid arthritis. Reproduced with permission [33] Copyright 2022, Elsevier. (**B**) In vivo imaging of the hind limb of an osteoarthritis mouse [44]. (**C**) The in vivo pharmacodynamic evaluation of drugs for rheumatoid arthritis was conducted by observing the clinical symptoms and bone contours of mice using micro-CT [44]. (**D**) The histopathological analysis of drugs for rheumatoid arthritis shows that the number of inflammatory cells has decreased and the degradation of cartilage matrix has been interrupted. Reproduced with permission [44] Copyright 2019, American Chemical Society.

JIA is a chronic childhood joint disorder, whose pathogenesis involves synovial inflammation, excessive production of ROS, and infiltration of immune cells. These three pathological processes interweave with each other, forming a self-amplifying malignant cycle within the developing joints of children, ultimately leading to erosion of joint cartilage, abnormal bone remodeling, and even lifelong disability. It is important to note that all current preclinical studies of nanozyme interventions in arthritis have used adult mouse/rat models of collagen-induced arthritis (CIA) or adjuvant arthritis (adult-onset rheumatoid arthritis RA models), and there is no evidence of pediatric-specific nanozymes efficacy in young animals or children with JIA. The current conclusion of nanozymes anti-arthritis is extrapolated from adult RA animal models to JIA people, and the interpretation should take into account the development stage of children’s immune system, the activity of joint growth plate, and the differences in pharmacokinetics/toxicity of nanomaterials during the development period. Firstly, nanozymes can directly intervene in the functional state of immune cells by eliminating ROS, thereby reversing the pro-inflammatory immune microenvironment into a pro-healing phenotype. On one hand, some researchers have found that MnO_2_ nanozymes can not only simulate the activities of SOD and CAT to eliminate ROS, but also regulate macrophages to polarize from the pro-inflammatory M1 type to the anti-inflammatory M2 type, reshaping the arthritis microenvironment [42]. The efficacy verification of this MnO_2_ nanozyme was completed in an adult mouse CIA model, which is extrapulatory evidence from the adult RA model and has not been replicated in JIA or young arthritis models. In addition, apart from innate immune cells, nanozymes can also regulate adaptive immunity. For example, using ultra-small Prussian blue nanoparticles functionalized with neutrophil exosomes (uPB-Exo), their inflammatory chemotactic properties are used to target and deliver to inflamed joints (Figure 4). This system eliminates ROS and regulates the balance between pathogenic Th17 cells and regulatory T cells (Treg), significantly reducing joint damage and inflammation, correcting immune disorders at the adaptive immune level [33]. Moreover, to cope with severe tissue damage in advanced arthritis, nanozymes have been innovatively applied in tissue engineering, creating a suitable regenerative microenvironment for stem cell therapy. For example, combining nanozyme-enhanced hydrogels with mesenchymal stem cells and implanting them into the joint, the nanozymes can eliminate ROS while catalyzing the production of oxygen, alleviating local hypoxia, effectively protecting stem cells from damage in the inflammatory microenvironment, and promoting cartilage repair and bone integration [43]. The nanozyme–hydrogel/stem cell system was validated in an adult rat model of osteochondral defects or OA, which was also an extrapolation of the adult model with no data from JIA or developmental joint models. In the future, it is urgent to establish a juvenile collagen-induced arthritis model (juvenile CIA) or transgenic JIA mouse model, and carry out age-stratified safety and intra-articular retention evaluation to confirm the intervention value of nanozymes in the real pediatric evidence chain for JIA secondary joint damage.

### 3.6. Inflammatory Bowel Disease (IBD)

**Figure 5:**
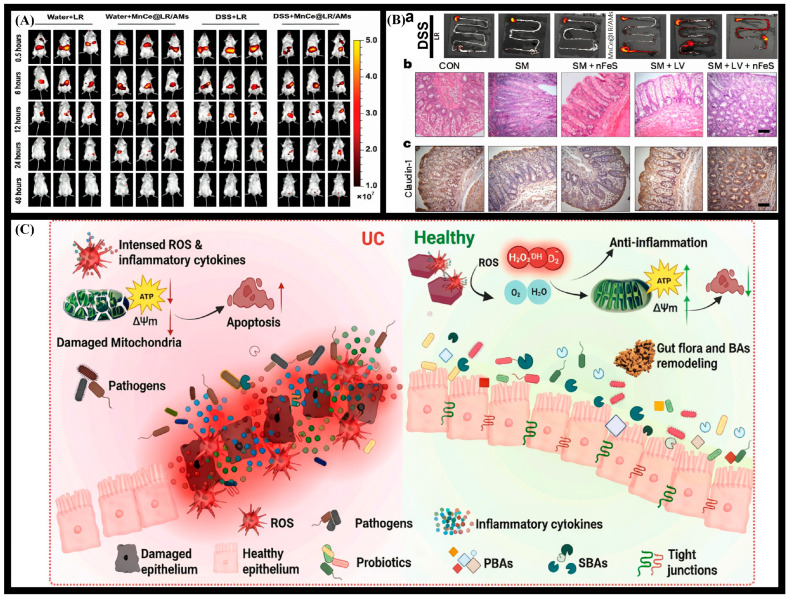
(**A**) In vivo imaging of rats with ulcerative colitis (UC) to evaluate the inflammatory targeting of Mn@CeO_2_ nanozymes. Reproduced with permission [49] Copyright 2025, American Chemical Society. (**B**) Fluorescence imaging of the intestine was performed with intestinal histopathological staining to assess inflammatory cell infiltration and intestinal epithelial integrity. Reproduced with permission [48] Copyright 2025, Springer nature. (**C**) Schematic diagram of antioxidative nanozymes–fucoidan embedded nanoclay gel mediated mitochondrial energy recovery and bile acid balance against ulcerative colitis. Reproduced with permission [35] Copyright 2025, Elsevier.

IBD, including ulcerative colitis (UC) and Crohn’s disease (CD), is a group of chronic, recurrent, and non-specific inflammatory disorders of the intestinal tract. Among children, the incidence of IBD is increasing year by year, and compared with adults, children with IBD often have a wider lesion range, higher disease activity, and more prominent extraintestinal manifestations such as growth retardation. At present, all the preclinical evidence for the efficacy of nanozymes in IBD intervention comes from adult mice (DSS or TNBS-induced UC/CD model) or adult rats, and there is no pediatric-specific efficacy data of nanozymes in young animals or children with pIBD. The current conclusion of nanozymes anti-IBD is based on the results of adult animal experiments to pediatric IBD population. It is necessary to consider the intestinal length/surface area ratio, mucosal immune development, the influence of growth plate, and the individual differences in absorption/retention of oral nanomaterials during the development period. The pathogenesis of IBD is complex and involves genetic susceptibility, environmental factors, and immune response disorders. Specifically at the pathological physiological level, intestinal oxidative stress, dysbiosis of the microbiota, and barrier damage are closely intertwined, forming the core pathological triangle that drives the occurrence and development of pediatric IBD. In response to this complex pathological network, nanozymes, with their unique nanoscale effect and the catalytic activity of mimicking natural antioxidant enzymes, can simultaneously act on the above three core links, demonstrating the potential of “multi-target synergy” treatment that traditional anti-inflammatory drugs or single biological agents cannot achieve. For example, nanozymes with SOD, CAT, and GPx mimicking activities can efficiently eliminate ·O_2_^−^, H_2_O_2_, and ·OH, thereby breaking the vicious cycle of “inflammation-ROS-tissue damage”. The effects of Mn_3_O_4_, CeO_2_, and MPBZs nanozymes on ROS clearance, barrier repair, and microbiota regulation were validated in an adult mouse DSS/TNBS-induced colitis model, which is a generalization from adult/adult animal IBD systems and has not been reproduced in pediatric or young IBD animal models. This broad-spectrum and efficient ROS clearance ability is a significant advantage of nanozymes over traditional small molecule antioxidants [34,45,46]. In addition, nanozymes can effectively protect mitochondrial function by clearing excessive ROS around epithelial cells and reduce epithelial cell apoptosis. More importantly, multiple studies have confirmed that nanozyme treatment can significantly upregulate the expression of tight junction proteins (such as Occludin, ZO-1, Claudin) and repair the damaged intestinal epithelial barrier. The restoration of barrier integrity not only reduces the leakage of harmful substances (such as bacterial endotoxins) but also blocks the continuous stimulation of the mucosal immune system by antigen substances (Figure 5) [35,47]. More importantly, nanozymes also indirectly create favorable conditions for the restoration of the balance of the intestinal microbiota by reshaping the redox microenvironment. Excessive oxidative stress environment is conducive to the excessive proliferation of aerobic pathogenic bacteria (such as certain Proteobacteria) and is not conducive to the colonization of anaerobic beneficial bacteria (such as Bifidobacterium and Lactobacillus). Nanozymes clear excessive ROS in the intestinal cavity and on the mucosal surface, reduce the local redox potential, and help restore the microenvironment conducive to the growth of beneficial bacteria, thereby indirectly promoting the correction of dysbiosis [48,49].

### 3.7. Necrotizing Enterocolitis (NEC)

**Figure 6:**
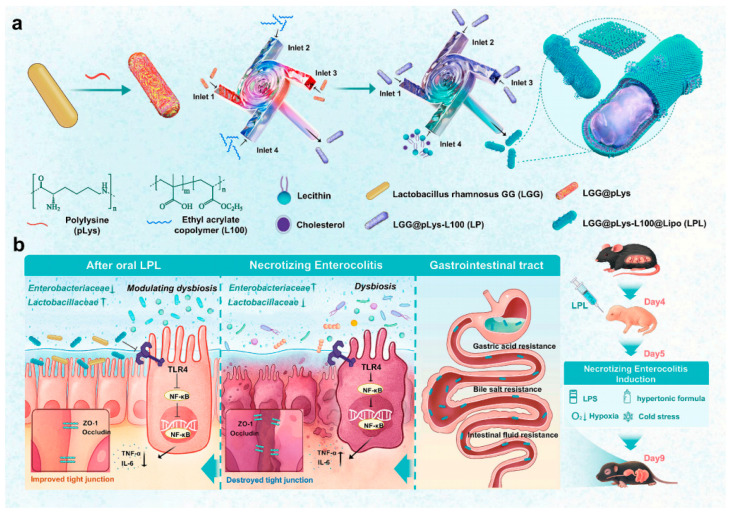
Schematic illustration of flash nano-encapsulation (FNE) of *Lactobacillus rhamnosus* GG (LGG) (**a**) and its mechanism for necrotizing enterocolitis (NEC) prevention (**b**) [51] Copyright 2025, Elsevier.

Necrotizing enterocolitis (NEC) is the most common and fatal gastrointestinal emergency in premature infants. Its core pathological mechanism involves explosive oxidative stress and excessive inflammatory response caused by intestinal ischemia–reperfusion, while the existing treatment methods are extremely limited. Nanozymes, with their antioxidant enzyme activities such as SOD and CAT, can efficiently eliminate excessive ROS at the lesion site, inhibit the TLR4/NF-κB inflammatory pathway, and protect the integrity of the intestinal epithelial barrier, providing a brand-new intervention idea for the drug treatment of NEC. Recently, the potential of naturally derived nanoscale bioactive vectors in the prevention and treatment of NEC has attracted much attention. In their review, Mahala et al. pointed out that exosomes derived from breast milk and cow’s milk, as natural nanovesicles, carry signaling molecules such as microRNAs, proteins, and lipids, which can resist degradation by gastrointestinal digestive enzymes and be effectively taken up by intestinal epithelial cells [50]. Several animal experiments have confirmed that human milk exosomes can significantly reduce the incidence and severity of experimental NEC in rats by 11.9–29%. The mechanism involves promoting intestinal epithelial cell proliferation, inhibiting apoptosis, and reducing inflammatory response. In addition, milk-derived exosomes also have low immunogenicity, high biocompatibility, and potential as a nano-carrier for chemotherapy drugs, providing a new platform for drug delivery in NEC. At the same time, probiotics, as a strategy to prevent NEC by regulating intestinal microecology, have shown potential to reduce the risk of NEC in preterm infants in several clinical trials. However, the survival rate of oral probiotics in the harsh environment of the gastrointestinal tract is extremely low, and the ability of oral probiotics to colonize the intestinal tract is limited, which seriously limits their clinical efficacy. In response to this bottleneck, Huang et al. recently reported an innovative flash nanoencapsulation technology to achieve efficient, continuous, and scalable single-cell nanoencapsulation of Lactobacillus rhamnosa GG by means of a multi-inlet vortex mixer [51]. As shown in the Figure 6, this technique uses a polyelectrolyte complex (poly-lysine and ph-responsive anionic polymer) as the first coating layer, and a phospholipid cholesterol lipid bilayer as the second coating layer to form a double shell structure about 300 nm thick. In vitro experiments showed that the survival rate of the double-shell encapsulated LGG was increased by 2 to 4 orders of magnitude compared with that of bare bacteria in simulated gastric juice, intestinal juice, and bile salt environments. In the neonatal mouse model of NEC, the survival rate of the LGG group was 84.6%, which was significantly lower than that of the NEC control group (53.8%), and the pathological damage score of intestinal tissue was significantly reduced. The protective mechanism of LGG was related to the inhibition of the TLR4/NF-κB signaling pathway and the up-regulation of tight junction proteins ZO-1 and Occludin, as well as to the remodeling of the composition of gut microbiota by increasing the abundance of beneficial bacteria Lactobacillus and reducing the abundance of pathogenic bacteria Enterobacteriaceae.

However, the transition from laboratory proof of concept to clinical practice in NICU still faces multiple bottlenecks. The extremely immature liver and kidney function and fluctuating hemodynamics of preterm infants lead to the highly unpredictable absorption, distribution, metabolism, and clearance of nanozymes. Due to the weak intestinal barrier and systemic immune system, nanozymes may cause immunogenic risks such as complement activation, protein corona formation, and amplification of non-specific inflammation. In addition, there is a lack of available experience in drug delivery devices for very low birth weight infants, sterile manufacturing processes, and ethical review and regulatory pathways for the neonatal population. Whether they are synthetic nanozymes, natural milk-derived exosomes, or nanoencapsulated probiotics, their application in the treatment of pediatric NEC faces common translational dilemmas: developmental dependence of pediatric pharmacokinetics, lifelong effects during critical windows of developmental toxicology, regulatory gaps in pediatric nanomedicins, and systematic engineering challenges for GMPS scale production. In the future, developmental stage-specific animal models and micro-sampling techniques are needed to clarify the pharmacokinetic characteristics of nanozymes in preterm infants, and biomimetic coatings or gut-targeted modifications are needed to reduce immune recognition. Only then can this emerging strategy truly benefit infants with NEC.

From the above application examples, it can be seen that nanozymes, through their multi-enzyme mimicking activity, intelligent response characteristics, and precise targeting ability, can effectively intervene in specific pathological processes of different children’s inflammatory diseases. Future research will focus more on improving the biological safety and disease specificity of nanozymes and on developing more child-friendly formulations, in order to accelerate their clinical transformation and benefit a large number of children.

## 4. Nanozymes for Multi-Modal Imaging-Guided Collaborative Therapy

In the traditional approach to treating pediatric inflammatory diseases, diagnosis and treatment are often conducted separately. This can result in problems like delays in treatment, challenges in monitoring how well a treatment is working, and sometimes unnecessary medical interventions. However, the development of nanozymes particles designed with specific catalytic functions that can also respond to the unique characteristics of disease environments promises to change this model. Nanozymes offer a path towards integrated diagnosis and treatment, allowing for a more cohesive and effective approach. This chapter will explore how nanozymes are applied in treating pediatric inflammatory diseases, focusing on their mechanisms, design strategies, and recent advancements in these innovative areas.

### 4.1. Multi-Modal Imaging-Guided Collaborative Therapy

#### 4.1.1. Multi-Modal Imaging

Nanozymes have shown significant promise in treating inflammatory diseases in children. These nanomaterials possess both catalytic properties and imaging capabilities, allowing them to act as safe and effective multimodal contrast agents. They can accurately identify small or deep inflammatory lesions in children’s bodies using techniques like magnetic resonance imaging (MRI), photoacoustic imaging (PA), or ultrasound imaging. This capability is especially crucial for young patients who may struggle to communicate their symptoms, as it enables early diagnosis and prompt intervention. During the treatment process, nanozymes can monitor the distribution and enrichment of drugs in the body in real time, dynamically feedback the release behavior of drugs in the lesion area, and assist clinicians in precisely regulating the dosage and treatment plan while protecting the delicate organs of children. Following treatment, nanozymes can facilitate non-invasive assessments of therapeutic effectiveness. By monitoring variations in imaging signals, they provide an objective measurement of inflammation resolution and tissue repair, minimizing the need for repeated invasive procedures or sedation that can be distressing for children. This multifunctional approach throughout the treatment journey addresses the critical need for precise, safe, and minimally invasive solutions for pediatric patients with inflammatory diseases. Additionally, it opens up new avenues for advancing pediatric precision medicine.

**Figure 7:**
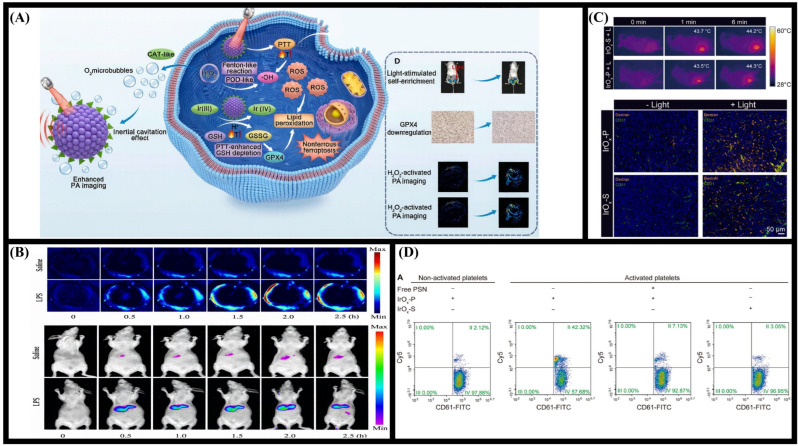
Visual localization of inflammatory sites mediated by ultrasound/photoacoustic imaging. (**A**) Schematic representation of the reaction mechanisms of nanozymes in the tumor microenvironment. Reproduced with permission [57] Copyright 2025, Elsevier. (**B**) Macrophage membrane-coated Cu–WO_3_–x-Hydro820 nanoreactor for treatment of inflammatory liver tissue and photoacoustic/fluorescence dual-mode imaging as in vivo pharmacodynamic evaluation of nanozymes. Reproduced with permission [58] Copyright 2024, American Chemical Society. (**C**) Imaging and targeted validation of nanozymes in small animals [57]. (**D**) The immune activation mechanism of nanozymes was evaluated through flow cytometry. Reproduced with permission [57] Copyright 2025, Elsevier.

**Figure 8:**
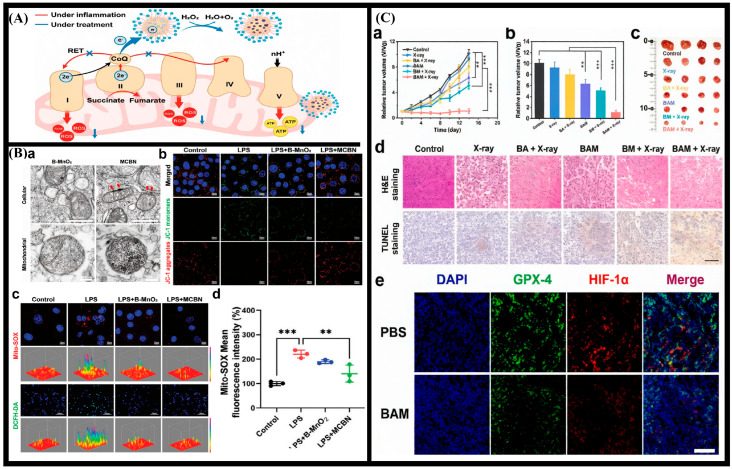
Nanozymes enhance ROS-mediated therapy by interfering with the electron transport chain. (**A**) Schematic representation of the mangium-based artificial mitochondrial complex “VI” as an electron and free radical conversion factory interfering with the electron transport chain and inhibiting the inflammatory response of macrophages. Reproduced with permission [59] Copyright 2024, Wiely. (**B**) DCFH-DA and Mito-SOX probes were used to detect intracellular ROS and mitochondrial ROS expression levels [59]. (**C**) The self-cascade nanohybrid enhanced the in vivo pharmacodynamic and histopathological evaluation of tumor radiosensitization therapy, including tumor volume measurement and immunofluorescence staining of ferroptosis-related markers. Reproduced with permission [58] Copyright 2022, Elsevier. Comparison of two different groups. *** *p* < 0.001, ** *p* < 0.01.

(1) Visualization and localization of inflammatory sites mediated by ultrasound/photoacoustic imaging. Many nanozymes themselves possess catalytic activity similar to CAT, which can catalyze the decomposition of excessive H_2_O_2_ produced at tumor or inflammatory sites to generate oxygen. This process can be utilized to enhance various imaging modalities, thereby achieving precise and non-invasive visualization of the lesion (Figure 7). In the fields of ultrasound and photoacoustic imaging, this catalytic oxygen production characteristic provides a unique molecular basis for the visualization and localization of inflammatory sites—the oxygen microbubbles generated in situ can serve as an acoustic enhancer for ultrasound imaging and significantly enhance the photoacoustic imaging signal through cavitation effects, thereby enabling high-sensitivity and high-resolution imaging of inflammatory lesions. For example, in the research on ultrasound-mediated visualization of inflammation, Yang et al. discovered in 2012 that Prussian blue nanoparticles (PBNPs) have CAT-like activity in neutral environments and can catalyze the decomposition of H_2_O_2_ to produce oxygen. When the oxygen concentration exceeds the saturation level, free bubbles can form, thereby achieving dual-mode imaging with ultrasound and magnetic resonance in vitro and in inflammatory animal models [52]. Recently, Zhao et al. designed pH-responsive PEG-SH and imidazole-modified gold nanoparticles (PMIZ-AuNPs), and this nanozyme aggregates in acidic inflammatory microenvironments and exhibits dual catalytic activities similar to SOD and CAT. In the congenital hydronephrosis and renal fibrosis mouse model, PMIZ-AuNPs can effectively accumulate at the damaged site and significantly enhance the ultrasound signal intensity, enabling real-time ultrasound monitoring of pediatric renal fibrosis inflammation [53]. In the field of photoacoustic imaging-mediated inflammation visualization, Zhao et al. developed Cu-WO_3x_-Hydro820 nano-reactors coated with macrophage membranes (CWHMs). This nano-reactor can target inflammatory tissues and utilize ROS (H_2_O_2_ and ·OH) at the inflammatory site to react with Hydro820 to form the near-infrared fluorescent group IR820, enabling dual-mode photoacoustic/fluorescence imaging of inflammatory liver tissue. Meanwhile, the Cu-WO_3x_ core of PMIZ-AuNPs has CAT-like activity and can catalyze the decomposition of H_2_O_2_ to alleviate oxidative stress [54]. Huang et al. constructed a biomimetic nanozyme probe BHTZ (BSA@HRP@TMB@ZIF-8) based on metal–organic frameworks (MOFs). This probe retains the three-dimensional structure and high catalytic activity of natural enzymes through in situ self-assembly of MOFs, and can activate the near-infrared absorption signal in the 1030 nm region under the presence of H_2_O_2_, achieving dynamic monitoring of ultra-trace H_2_O_2_ in mice with drug-induced liver injury, demonstrating the potential application in the precise diagnosis of inflammatory diseases [55]. Additionally, Chen et al. revealed IrO_x_-P nanozymes that not only have CAT-like activity and can generate O_2_ microbubbles in the presence of H_2_O_2_, but also enhance the photoacoustic signal through non-inert cavitation effects, enabling real-time imaging monitoring of the catalytic treatment process. These studies collectively indicate that the oxygen generation strategy based on the CAT-like activity of nanozymes provides a universal platform for ultrasound and photoacoustic imaging-mediated inflammation visualization and localization, and is expected to promote the early diagnosis and integrated diagnosis and treatment of inflammatory diseases [56].

(2) Localization of inflammatory sites mediated by magnetic resonance imaging (MRI). The contrast of MRI depends on paramagnetic substances (such as Gd^3+^ and Mn^2+^). Some nanozymes undergo structural changes when they exert CAT activity, generating paramagnetic substances in situ, thereby achieving “intelligent activation” type MRI. For example, manganese (III) oxide (Mn_3_O_4_) nanozymes decompose in an acidic, high H_2_O_2_ inflammatory microenvironment, generating paramagnetic Mn^2+^, which can be used as a contrast agent for T1-weighted MRI, making the inflammatory areas “brighter” on the image. This “activated” imaging ensures a positive correlation between the signal and the pathological degree, achieving high-contrast, real-time visualization of inflammation. Researchers have found that by utilizing the property of MnO_2_ decomposing into Mn^2+^ in an inflammatory environment, MnO_2_ nanozymes are intravenously injected into arthritis model mice. The nanozymes will accumulate at the inflamed joint synovium through “enhanced permeability and retention effect”. It was found that, in the inflammatory joints, Mn^2+^ generated by the reaction between MnO_2_ and H_2_O_2_ is an excellent T1-weighted MRI contrast agent. Therefore, only in the inflamed joints will the MRI signal significantly enhance, while normal tissues will not show obvious signals (Figure 8) [59]. This “switch” type imaging mode greatly improves the specificity and accuracy of diagnosis. This method avoids the possible systemic toxic side effects (such as renal systemic fibrosis) caused by traditional Gd-based contrast agents in children, and realizes the complete synchronization of treatment and diagnosis.

For pediatric patients, this kind of integrated diagnostic and therapeutic nanozymes, with its ability to provide visual targeting, offers some real advantages. For one, both ultrasound and MRI are non-invasive imaging methods. They do not require endoscopic procedures, so there is less trauma, which makes them much more suitable for children. Second, these nanozymes can clearly show where the inflamed areas are, helping doctors give local treatment or check how well the therapy is working. This avoids the harm that can come from giving medication throughout the whole body in a child. Finally, the “smart activation” feature means the contrast agent stays quiet in healthy tissues, which reduces non-specific binding and potential toxicity. This gives a solid basis for tailoring medication to each child’s needs.

#### 4.1.2. Self-Reporting Nanozymes System

Such systems go even further, as the signals they generate directly reflect the intensity or outcome of the treatment response, representing a higher level of integrated diagnosis and treatment.

Firstly, the signal output accompanying the catalytic reaction is an advanced form of integrated diagnosis and treatment. The core lies in the fact that, when nanozymes perform their catalytic functions, their own physical and chemical properties (such as oxidation state, crystal structure, electronic state) will undergo regular changes. These changes can directly generate or modulate optical, magnetic, or acoustic signals. This enables doctors or researchers to assess the distribution, activation status, and therapeutic efficacy of nanozymes in real time and in situ without introducing exogenous reporter molecules. For example, when CeO_2_ nanozymes eliminate ROS such as ·O_2_^−^, their surface Ce^4+^ will be reduced to Ce^3+^, while generating a positively charged oxygen vacancy. This change in oxidation state and surface defect will drastically alter the collective oscillation behavior of the internal free electrons, namely the localized surface plasmon resonance (LSPR) effect. As the proportion of Ce^3+^ increases, its LSPR absorption peak may shift either redward or blueward, and the absorbance intensity will change. By observing the color change in the solution using a spectrometer or even with the naked eye, the ROS clearance ability and treatment progress can be semi-quantitatively evaluated [60]. The main mechanism is that the 4f electron configuration of Ce^3+^ enables it to undergo f-f or f-d transitions, thereby generating intrinsic fluorescence, while Ce^4+^ is usually a fluorescence quencher. Therefore, when CeO_2_ nanozymes are involved in the antioxidant process, the proportion of Ce^3+^ on the surface increases, and its fluorescence intensity will correspondingly increase. Through a fluorescence spectrometer or a small animal in vivo imaging system, the dynamic antioxidant treatment of nanozymes at the inflammatory site in the animal body can be monitored in real time and non-invasively, visually demonstrating the correlation that “the more effective the treatment, the stronger the fluorescence”.

Furthermore, the intelligent sensing and therapeutic system based on FRET (Fluorescence Resonance Energy Transfer) represents a sophisticated frontier in the field of nanozymes research. It ingeniously couples high sensitivity diagnosis with precise on-demand treatment on the same nanoscale platform. The main mechanism: taking human neutrophil elastase (hNE) as an example. This protease is elevated in various inflammatory diseases. When the device enters the inflammatory microenvironment, hNE will specifically cleave the peptide chain linker connecting two fluorescent molecules. The cleavage disrupts the FRET effect, restoring the donor fluorescence, thereby outputting a strong and detectable fluorescence signal, achieving ultra-sensitive detection of inflammatory markers. The generation of the above fluorescence signal is not only used to report the disease state but can also be used as a visual basis for initiating treatment in practice. In the conceptually advanced system, the diagnostic phase directly or indirectly activates the therapeutic module. During or after the sensing process, the therapeutic module is activated. For example, a research team from Peking University and Nankai University developed a “AND” logic gate FRET/magnetic resonance tuned (DRET) nanoprobe [61]. The design of this probe is very ingenious, aiming to simultaneously monitor two key immune-related biomarkers: the granzyme B (GrB) released by activated CD8+ T cells and the aspartate proteolytic enzyme-3 (Caspase-3) produced by apoptotic tumor cells. Only when GrB appears will the fluorescence signal that was previously FRET-quenched be restored; only when Caspase-3 appears will the T1 signal of magnetic resonance imaging (MRI) be enhanced. This “AND” logic gate design enables the observation of dual changes in fluorescence and MRI signals only when T cells are effectively activated and tumor cells do indeed undergo apoptosis. This strategy can more accurately evaluate the actual effect of anti-tumor immunity and even predict the efficacy before tumor volume reduction and identify drug resistance phenomena.

### 4.2. Synergistic Therapy

The pathogenesis of pediatric inflammatory diseases is complex, and a single therapy often has limited effectiveness. Integrating drugs with nanozymes enables the complementary and enhanced efficacy of catalytic treatment and chemical treatment mechanisms, achieving more precise, powerful, and targeted treatment. This strategy is particularly applicable to pediatric inflammatory diseases with complex pathogenesis and the need for precise medication, representing an important direction for the future development of pediatric nanomedicine.

#### 4.2.1. Catalytic and Chemical Therapy

Nanozymes can not only catalyze treatment by themselves, but also act as intelligent carriers to optimize the delivery and efficacy of traditional chemical drugs. Both quantitatively and mechanistically, the effectiveness of the nanozymes–drug synergy system can be systematically elucidated by drug loading performance, microenvironment responsive release kinetics, catalytic reaction rate constant, and pharmacodynamic parameters at the cell/animal level. On one hand, loading mitochondrial-targeted drugs (such as triphenylphosphine, TPP) onto nanomachines enables a “1 + 1 > 2” strategic design. The synergistic effect mainly manifests in two aspects. Firstly, the physical synergistic delivery using the size effect and surface modification of nanozymes is to achieve targeted enrichment at the inflammatory site. More importantly, the biological functional synergy, that is, the catalytic activity of nanozymes and the pharmacological mechanism of the drug, cooperate with each other to jointly break the vicious cycle of the disease. For example, Yang et al. loaded TPP onto MnO_2_ nanomachines for the treatment of periodontitis [58]. The MnO_2_ nanozyme themselves have good biocompatibility and can achieve preferential aggregation at the gingiva inflammatory site through surface modification (such as connecting targeting peptides) or the enhanced permeability and retention effect (EPR effect) of inflammatory tissues. The core advantage of this system lies in eliminating ROS and alleviating hypoxia, reversing the pro-inflammatory microenvironment, which is equivalent to creating a more favorable “battlefield” for TPP to function, significantly enhancing the sensitivity of cells to TPP and overcoming the common drug resistance in inflammatory environments. At the same time, MnO_2_ nanozyme indirectly promote the expansion and function of regulatory T cells (Treg) and inhibit pathogenic Th2 and Th17 cells. This parallel action at different targets and ultimate convergence towards immune homeostasis remodeling is the fundamental reason for achieving a powerful synergistic therapeutic effect. Due to its targeting and synergistic effect, while achieving the same or better therapeutic effect, the systemic exposure dose of TPP can be significantly reduced, thereby reducing its potential side effects on children’s growth and development (such as height growth and hormone levels). Moreover, in the treatment of abdominal infections or sepsis (both can trigger systemic inflammatory responses), researchers combined antibiotics (such as vancomycin) with nanozymes with POD activity (such as Fe_3_O_4_) [62]. The Van loading capacity of Fe_3_O_4_ nanozymes (~20 nm) was about 68 μg/mg, and the VAN release rate was about 3.5 times higher under pH 5.0 than that under pH 7.4. Mechanologically, Fe^2+^/Fe^3+^ redox pair on Fe_3_O_4_ surface catalyzes H_2_O_2_ through the Haber–Weiss cycle to produce highly toxic ·OH (EPR spin capture quantification showed that the signal intensity of ·OH was 6.3 times that of the H_2_O_2_ alone group) under weakly acidic conditions. ·OH attack on the bacterial molecular layer caused lipid peroxidation (2.9-fold increase in MDA content), leading to a dramatic increase in membrane permeability (about 4-fold increase in DiSC_3_(5) depolarization fluorescence), which greatly promoted Van molecules into the bacterial membrane. Van inhibits peptidoglycan cross-linking and interacts with ·OH to form an inner and outer clamp. The Fe_3_O_4_ nanozyme can catalyze the production of highly toxic ·OH in the weakly acidic infection microenvironment, which causes damage to the bacterial cell membrane, greatly promoting the efficiency of vancomycin molecules entering the bacterial interior. Vancomycin then inhibits cell wall synthesis, forming an internal and external counterattack with the ·OH’s “breaking the wall” effect. The ·OH produced by the nanomachine can effectively disintegrate the bacterial biofilm, exposing the “retained bacteria” hidden inside to the antibiotic, thereby curing chronic infections and eliminating the source of inflammation. Through rapid and effective clearance of pathogens, it can prevent the uncontrolled escalation of systemic inflammatory responses and avoid the progression of sepsis to a severe stage, which is crucial for children with an immature immune system.

On the other hand, achieving self-supply of H_2_O_2_ to enhance chemical kinetics therapy is centered on using Fenton or similar Fenton reactions to convert intracellular low-toxicity H_2_O_2_ into highly toxic hydroxyl radicals, thereby selectively killing diseased cells. However, its efficacy is severely limited by the insufficiency of endogenous H_2_O_2_ in tumor or inflammatory microenvironments. Additionally, direct exogenous supplementation of H_2_O_2_ poses safety risks and is difficult to control. The “self-supply H_2_O_2_” strategy emerged as a solution. It achieves this through ingenious nano-design, allowing the therapeutic system to generate H_2_O_2_ on-demand and in situ at the lesion site, thereby significantly enhancing the catalytic kinetics effect. Research has constructed a system that encapsulates Pd nanozymes and camptothecin (CPT) [57]. The encapsulation efficiency of CPT by this Pd@MSN vector was 91.2 ± 2.8%, and the drug loading was 8.7 wt%. Mechanistically, CPT induces DNA damage through the stabilization of the topoisomerase I-DNA complex, activates NADPH oxidase (NOX2/NOX4) activity, and increases the intracellular H_2_O_2_ level from about 60 μM at baseline to about 280 μM (fluorescence probe Amplex Red). The POD-like Pd nanozymes could efficiently produce ·OH through Fe^2+^/Pd^2+^ -mediated Fenton reaction (·OH yield was increased by about 4.6-fold compared with the CPT without treatment, and DCF fluorescence intensity was increased by about 5.2-fold), amplify oxidative stress, and synergistically induce tumor cell apoptosis (apoptosis rate increased from 23% to 61% in CPT-alone treatment). Here, CPT can activate NADPH oxidase, increasing the H_2_O_2_ level within the tumor, thereby “fueling” the supply of Pd nanozymes with POD activity, enabling them to efficiently produce ·OH, amplifying oxidative stress, and synergistically inducing tumor cell apoptosis. This strategy is also applicable to eliminating pathogens or regulating abnormal proliferating immune cells. Although these cases originated from tumor treatment, the “self-supply H_2_O_2_” strategy to enhance catalytic kinetics has great potential in stubborn inflammatory diseases, especially in scenarios where excessive activated immune cells or pathogens need to be cleared. In chronic inflammation such as rheumatoid arthritis, there are a large number of pro-inflammatory M1-type macrophages in the joint synovium, which drive the persistence and development of inflammation. Researchers have combined GPx-loaded manganese-based nanozymes with macrophage-targeting peptides, and the nanosystem is engulfed by M1 macrophages in large quantities through the targeting peptide [63]. MnO_2_ nanozymes have a particle size of about 45 nm and a GOx loading of 12.3 wt%. In the pH 6.8 inflammatory microenvironment, MnO_2_ partially solubilizes to release Mn^2+^ and expose the Fenton-like active center. Cellular uptake assay showed that M2pep-modified M1 macrophages increased the uptake of the nano-system by about 3.8-fold compared with the non-targeted group (confocal quantification). Inside the cells, glucose is catalyzed by GOx to be oxidized into gluconic acid and produce H_2_O_2_. The manganese-based nanozyme uses the produced H_2_O_2_ to explosively generate ·OH through a Fenton-like reaction, and the high level of ·OH will induce apoptosis or ferroptosis in the over-activated M1 macrophages, specifically reducing the pro-inflammatory cell population without affecting normal immune cells, potentially fundamentally changing the immune microenvironment of the joint and achieving long-term remission. The “self-supply H_2_O_2_” strategy integrates the functions of fuel generation and toxic product production into a single nano-platform, perfectly solving the bottleneck problem of CDT and achieving self-amplification of catalytic kinetics.

#### 4.2.2. Catalytic Therapy and Phototherapy

Although nanozymes have powerful functions, if their activity is constantly activated within the body, it may also cause unnecessary effects on normal tissues. By utilizing physical energies such as light and sound, “on-demand activation”, “activation at specific sites”, and “control of activation intensity” can be achieved. This significantly enhances the safety and accuracy of treatment, and is particularly suitable for children patients who have high requirements for treatment precision.

(1) Phototherapy–Catalytic Synergistic Therapy. Many nanozymes (such as Prussian blue, Pd nanozymes) have excellent photothermal conversion efficiency. Under NIR irradiation, they can convert light energy into thermal energy. This photothermal effect itself can provide mild thermal therapy for inflammatory tissues, inhibiting overactive immune cells. At the same time, local heating can significantly accelerate the catalytic reaction rate of nanozymes and enhance the efficiency of ROS clearance or generation. Moreover, the thermal effect can also promote the intelligent release of loaded drugs. For example, JIA is characterized by chronic inflammation of the joint synovium, leading to cartilage and bone destruction. PBNZs achieve multiple benefits in the treatment of JIA through the synergistic effect of photothermal and catalytic processes [64]. It not only rapidly suppresses the rampant activity of inflammatory cells through physical heat therapy, but also fundamentally improves the pathological microenvironment of the joint cavity (clearing ROS, alleviating hypoxia) by enhancing the catalytic effect of heat, thereby promoting the transformation of macrophages to repair type and achieving long-lasting immune regulation. In addition, there is severe oxidative stress and barrier damage in the intestinal tract of patients with inflammatory bowel disease. Pt@PCN222-Mn ascade nanozymes are a well-designed bimetal nanozyme [65]. The nanozymes reach the inflammatory site in the colon through oral administration, and are irradiated with near-infrared light through colonoscopy or non-invasive methods. The Pt shell efficiently clears various ROS and directly alleviates oxidative damage; at the same time, the Pt core converts light energy into thermal energy. Local heating doubles the antioxidant enzyme activity of the Pt shell, reaching the peak efficiency in clearing ROS, and the combined treatment significantly reduces the levels of inflammatory factors, and due to the relief of oxidative stress and the improvement in local blood flow, promotes the proliferation and migration of intestinal epithelial cells, accelerates the recovery of tight junction proteins, and thereby repairs the damaged physical barrier of the intestinal tract.

(2) Sonodynamic–Catalytic Synergistic Therapy. For deep joint cavity inflammation in children (such as the hip joint involved in juvenile idiopathic arthritis), spinal inflammation or deep organ inflammation, traditional drugs have difficulty penetrating effectively, while surgery or repeated injections are traumatic and risky. The combined sonodynamic and catalytic therapy utilizes ultrasound as a control and tissue manipulation method, possessing unique advantages of being non-invasive, deep penetration, and precise focusing. Through synergistic action, it efficiently cleanses the lesion. The mechanism of action is summarized as follows: Ultrasound can safely and non-invasively penetrate tissues and precisely focus on deep inflammatory lesions. In the focused area, ultrasound energy activates the “sonosensitizer” (usually a special nanozyme) that has been pre-enriched here, initiating the treatment process. The activated sonosensitizer converts ultrasound energy into chemical energy, prompting the surrounding environment to convert oxygen into a large amount of ROS, especially singlet oxygen. These ROS can directly destroy over-activated immune cells or the cell membranes, proteins, and DNA of pathogens, inducing them to undergo immunogenic cell death—a type of death where cells die in a way that can send “alarm signals” to the body’s immune system when they are on the verge of death. At the same time, the GPx activity of the nanozyme itself is mobilized. The above processes jointly trigger ferroptosis—a type of iron-dependent cell death caused by the accumulation of lipid peroxides. The key point is that this is an immunogenic ferroptosis. This means that the dead cells will release more signals, strongly activating and “educating” the main immune forces such as T lymphocytes in the body, enabling them to recognize and monitor the inflammatory site for a long time and prevent recurrence, thereby achieving long-lasting immune protection.

For example, pediatric suppurative arthritis is a severe inflammation caused by bacterial infection of deep joints (such as the hip joint and knee joint), which can rapidly destroy cartilage and lead to lifelong disability. Traditional intravenous antibiotics have difficulty penetrating the joint synovial barrier and cannot solve the problems of bacterial biofilms and excessive inflammatory responses. Researchers have constructed a hollow MnO_2_ nanozyme with bacterial membrane mimicry, which is loaded with efficient photodynamic agents and has POD and CAT activities [44]. On one hand, the photodynamic effect directly physically destroys the stubborn biofilms protecting the bacteria, while the catalytic activity of the nanozyme, under the command of ultrasound, eliminates exposed bacteria and over-activated immune cells through the production of ·OH and consumption of GSH, and during the entire process, the immunogenic cell death and ferroptosis induced not only eliminate “bad cells”, but more importantly, activate the body’s own specific immunity, providing long-term protection against infection and preventing recurrence. The synergistic treatment strategy of photodynamic–catalysis, with its four core advantages of non-invasive deep penetration, precise temporal and spatial control, multiple mechanism synergy, and activation of long-lasting immunity, has opened up a new path for treating deep tissue inflammation in children that traditional therapies have been unable to address. It minimizes the trauma and side effects of the treatment on the growing body of children and represents an important development direction for personalized and precise pediatric treatment.

#### 4.2.3. Immunotherapy–Catalytic Therapy

Traditional anti-inflammatory treatments often focus on broad-spectrum inhibition of immune responses, which may lead to side effects such as decreased immune system function, and make it difficult to prevent disease recurrence. The core of the catalytic therapy and immunotherapy synergy strategy lies in utilizing the catalytic activity of nanozymes to not only directly eliminate inflammatory mediators but also actively and intelligently reshape the imbalanced immune microenvironment. Combining the short-term catalytic anti-inflammatory effect with the long-term immune memory protection, it achieves the “treatment of both symptoms and root causes” effect. The main mechanism is as follows: nanozymes first use their antioxidant activities such as SOD and CAT to efficiently eliminate inflammatory signal amplifiers, directly reducing tissue damage and oxidative stress. This step is like “putting out the fire”, creating a stable “soil” for the subsequent immune reconstruction. At the same time, on the basis of eliminating ROS and alleviating hypoxia, nanozymes directly intervene in the functions and fates of immune cells through multiple mechanisms, promoting the immune state to shift from “pro-inflammatory” to “anti-inflammatory/repair”, and possibly establishing immune memory to prevent recurrence. This step is like “improving the soil and sowing seeds”, achieving long-term stability.

For instance, in the later stage of inflammation or in some chronic autoimmune diseases, an immunosuppressive microenvironment will form locally at the lesion site. In this environment, the extracellular enzyme CD73 will hydrolyze the immune activation signal molecule ATP into the immunosuppressive molecule adenosine, thereby “paralyzing” the function of immune T cells. Studies have constructed MOF nanozymes [66] that are loaded with antisense oligonucleotides targeting CD73. This nanozyme has a Ru single-atom catalytic site. This system, on the one hand, efficiently decomposes H_2_O_2_ in the inflammatory area through the Ru single-atom site, alleviating tissue hypoxia, thereby inhibiting the HIF-1α signaling pathway (this pathway upregulates the expression of CD73. This is reducing the production of “paralyzing signals” at the upstream level. On the other hand, after the ASOs carried by the nanozyme enter the cells, they can directly downregulate the expression of the CD73 gene, fundamentally reducing the quantity of this “paralyzing enzyme”. More importantly, through the dual attack of “catalytic improvement of the environment” and “direct genetic intervention”, the adenosine level in the lesion area is significantly inhibited. The previously “paralyzed” T cells are reactivated, restoring their ability to kill pathogens and clear necrotic cells, thereby reversing the immunosuppression and rebuilding an effective immune surveillance. In addition, cholangitis induced by immune checkpoint inhibitors (ICIs) is a rare and serious pediatric immune disease that is closely related to immunotherapy. Although the application of PD-1 inhibitors such as nivolumab is increasing in pediatric malignancies, immune-related adverse events (irAEs) caused by nivolumab also need to be paid great attention. The retrospective study by He et al. showed that although the incidence of nivolumab-induced cholangitis was extremely low (approximately 0.73%), the clinical presentation was diverse and the prognosis was poor: The median time of onset was 117 days after treatment. The main manifestations were abdominal pain (42.1%) and fever (18.4%), and some patients (15.8%) only showed asymptomatic elevated liver enzymes [67]. Laboratory tests showed that the median value of alkaline phosphatase (ALP) was as high as 1721 IU/L, and the median value of γ-glutamyl transpeptidase (GGT) was 829 IU/L, suggesting that cholestasis was the main characteristic. Imaging examination showed intrahepatic and extrahepatic bile duct dilatation, hypertrophy, and stenosis. Liver biopsy was mainly CD8^+^T lymphocyte infiltration. These data suggest that ICI-induced cholangitis is characterized by insidious onset, rapid progression, and strong resistance to steroids, which is particularly harmful to children. Considering that children are in a critical period for the development of the immune system and hepatobiliary organs, once such adverse reactions occur, they may lead to irreversible bile duct damage or even disappear bile duct syndrome. Therefore, the particularity of this immune-mediated disease should be fully recognized when developing a nano-enzyme diagnosis and treatment platform for children. On the one hand, nano-enzymes can be used as a tool to remove ROS from the excessive inflammatory microenvironment and reduce CD8^+^T cell-mediated bile duct epithelial injury. On the other hand, surface modification strategies of nanozymes, such as conjugation of UDCA or ligands targeting bile duct epithelial cells, can also be used to achieve precise delivery to the site of bile duct inflammation and local immune regulation, thereby effectively controlling the progression of ICI-related cholangitis without affecting the systemic antitumor immune response. In the future, the combination of nano-enzyme technology and immune monitoring means may provide a new prevention and treatment strategy for rare but fatal cholangitis during ICI treatment in children.

#### 4.2.4. Biotherapy–Catalytic Therapy

Combining nanozymes with biological treatment technologies such as cells and genes is indeed one of the most cutting-edge exploration directions at present. The core of this collaborative strategy lies in “taking the strengths of one and compensating for the weaknesses of the other”—it can not only utilize the designed catalytic function of nanozymes to improve the complex in vivo environment faced by biological therapies, but also leverage the high specificity and long-lasting nature of biological technologies themselves to ultimately achieve a precise synergistic therapeutic effect.

(1) Combination of cell therapy and catalytic therapy: One of the major challenges faced by cell therapies (such as stem cell therapy and immune cell therapy) is that when the therapeutic cells are delivered into the body, the harsh microenvironment of the target tissue (such as inflamed, hypoxic joints or tumors) can significantly affect the survival and function of these cells. Nanozymes can act as “pioneers” and “logistics support” to protect these therapeutic cells. For example, in inflammatory and other diseases, researchers attempt to implant mesenchymal stem cells together with hydrogels carrying nanomachines into the joint cavity [68]. Here, the nanozymes mainly play two roles: eliminating ROS by utilizing their SOD and CAT activities and alleviating hypoxia by catalytic reactions. Thus, the nanozymes are protecting the stem cells, significantly improving the survival rate of the stem cells, enabling them to better perform the functions of repairing cartilage and regulating immunity. At the same time, Gao et al. designed an engineered nanomachine that can be activated by deep red light or ultrasound. Under the guidance of external energy, the nanozyme catalyzes the generation of a large number of artificial antigen “patches” on the surface of cancer cells. Subsequently, a bispecific T-cell linker molecule is introduced, which grabs the artificial “patches” at one end and firmly “holds” the T cells at the other end, directly guiding the body’s “immunological army” T cells to the tumor site and activating their “strongest attack mode” for killing [69]. This method ingeniously utilizes the catalytic ability of the nanomachine, solves the problem that immune cells have difficulty recognizing “deceptive” cancer cells, achieves precise tumor clearance, and is expected to induce long-term immune memory, similar to receiving a “tumor vaccine”.

(2) Combination of gene therapy and therapeutic catalysis: Gene therapy aims to correct erroneous genetic information at its root, but its development is limited by issues such as low delivery efficiency and immunosuppression in the tumor microenvironment. Nanozymes can play the roles of “enhanced delivery agents” and “microenvironment regulators” in this process. For instance, in an asthma model, a biomimetic nanosystem was used to deliver siRNA, successfully knocking down the estrogen receptor α (ERα) gene related to ferroptosis in airway epithelial cells, while the nanozymes themselves performed antioxidant functions, achieving the synergy of gene therapy and catalytic therapy. Another example is in the research on pancreatic cancer, where a dual-enzyme-responsive DNA nanomaterial developed by researcher Liu Ying’s team was used for the co-delivery of molecular beacons and the CRISPR-Cas9 gene editing system [70]. This system could release molecular beacons for specific fluorescence imaging within cancer cells and release CRISPR-Cas9 for gene therapy simultaneously. It can be envisioned that if nanozyme components are introduced into this system, such as using them to consume the excessive H_2_O_2_ present in the tumor microenvironment or alleviating hypoxia, it could create a more “friendly” working environment for gene editing tools, potentially enhancing the efficiency and therapeutic effect of gene editing. Through the dual strategies of “catalytic improvement of the environment” and “direct genetic intervention”, the generation of immunosuppressive molecule adenosine was significantly inhibited, thereby relieving the “paralysis” state of immune cells and activating anti-tumor immunity.

Overall, the synergy between nanozymes and biotechnology marks that nanozymes are moving from simple “drug treatment” to complex “cell/gene repair and regulation”. Nanozymes play multiple roles as enhancers, protectors, and navigators in this process. Of course, the clinical application of this cutting-edge field to mature still requires continuous exploration in aspects such as targeted precision, long-term biological safety (especially for children), and large-scale production. However, there is no doubt that this “strong alliance” strategy has opened up a promising future vision for us to overcome many complex diseases, including childhood inflammatory diseases and cancer.

## 5. Challenges and Prospects

### 5.1. Challenges of Clinical Translation

#### 5.1.1. Long-Term Safety and Biodegradability

On the one hand, the human body of children is in a dynamic process of rapid growth and metabolism. In comparison to adults, their organ systems are still developing, and their immune systems are not as strong. Their ability to repair and regenerate tissues also differs from that of adults. During this critical period, many inorganic nanomaterials can be challenging for the body to break down and remove effectively. As a result, these materials may linger in certain organs, such as the liver and spleen, where they could disrupt normal metabolic functions and potentially accumulate over time. As shown in the rat model, exposure to SiO_2_ nanoparticles during the neonatal period can cause excessive activation of microglia in the hippocampus and inhibit the proliferation of neural progenitor cells, suggesting that the passage of nanomaterials through the developing blood–brain barrier poses a risk of long-term neurobehavioral damage [71]. A significant concern is that if nanozymes cross the still-developing blood–brain barrier, they could cause harmful effects on children’s central nervous systems. Furthermore, the effect of these materials on the reproductive system which is also developing, has not been thoroughly studied for safety. It is crucial to evaluate the long-term compatibility, immune responses, and potential genetic damage caused by nanomaterials as we move from basic research to clinical use in pediatrics.

Furthermore, the interaction between nanomaterials and the developing immune system is complex and poorly understood. During childhood, especially in the early years, the immune system shifts from relying on “passive immunity” to developing active immune tolerance and memory. This critical phase means that nanomaterials could significantly impact how the immune system matures. They might change how antigen-presenting cells function, affect the growth of regulatory T cells, or alter the balance of gut bacteria. Specifically, a key concern is whether exposure to these materials could disrupt the normal shrinking of the thymus or upset the balance between different immune responses (Th1/Th2). This disruption could potentially increase the long-term risk of autoimmune diseases like type 1 diabetes or allergic conditions like asthma and eczema. This is a pressing issue that needs further investigation. Some studies have also shown that the co-exposure of TiO_2_ nanoparticles with antigens can upregulate the secretion of Th2-type cytokines such as IL-4 and IL-13, and aggravate airway allergic inflammation, suggesting that nanomaterials may increase the susceptibility to allergic diseases by disrupting the Th1/Th2 balance [72]. To address these risks, it is essential to go beyond short-term toxicity tests. A comprehensive long-term safety assessment is needed, considering the entire period of growth and development from infancy through childhood, adolescence, and into early adulthood. Important focus areas should include maintaining immune balance, the effectiveness of forming immune memory, and any potential effects on vaccine responses.

#### 5.1.2. Precise Dose and Individualized Administration

Firstly, the age range of children is wide, ranging from extremely premature infants (gestational age < 28 weeks) to adolescents in the late stage of puberty (18 years old). There are significant and dynamic individual differences in their weight, body surface area, organ maturity, and pharmacokinetic parameters for drug metabolism. For instance, the activity of liver drug-metabolizing enzymes (such as the CYP450 enzyme system) in the neonatal period is only 20–50% of that in adults, and the glomerular filtration rate does not reach the adult level until several months after birth; after entering puberty, the rapid changes in sex hormone levels may further affect the distribution and metabolism of drugs. Therefore, simply estimating the dosage based on adult or older children’s data is extremely unreliable. It may not only lead to ineffective treatment but also cause toxic reactions or delay the condition due to excessive or insufficient blood drug concentration. A stratified and precise dosimetry model based on multiple parameters such as age, corrected gestational age, weight, body surface area, and organ development index must be established, combined with pediatric physiological pharmacokinetic modeling and validation with real-world clinical data, to formulate individualized medication regimens that truly meet the needs of children at different developmental stages.

On the other hand, the pharmacokinetic studies of nanozymes in the pediatric field are currently almost completely lacking, which constitutes one of the most urgent knowledge gaps in the process of transitioning from basic research to clinical application. Specifically, children are not miniature adults. Their gastric pH values (neutral in newborns and gradually acidic in infants), pancreatic enzyme activity, bile secretion function, and gastrointestinal motility rhythms all change with the developmental stage, which directly affects the absorption efficiency and bioavailability of nanozymes administered orally. As research has shown, the slightly alkaline pH in the stomach of newborn mice results in different processes of the dissolution and micellization of lipophilic nanocrystals compared to adults, leading to a significantly higher bioavailability of oral nanocapsules in young individuals than in adult animals [73]. Among them, the secretion capacity of pancreatic enzymes and bile acids in infants is only 10–60% of that in adults, resulting in the micellilation and transmembrane transport efficiency of fat-soluble nanocarries (such as liposomes and PLGA nanoparticles). In addition, the high permeability of the neonatal intestinal barrier is conducive to the absorption of macromolecules, but it also increases the potential risk of non-specific nanoparticles into the blood. In the distribution stage, children have a higher proportion of blood volume relative to body weight and lower plasma protein levels, and their plasma protein concentration continues to rise during the first few months of life, resulting in dynamic changes in the plasma protein binding rate and free concentration of nanozymes. More importantly, the functions of the blood–brain barrier and blood–milk barrier are immature. The expression of tight junction proteins and the function of P-glycoprotein efflux increase with age. The immature BBB enables the PLGA-PEG nanoparticles to more easily penetrate and enter the brain parenchyma in the early postnatal period and remain in the brain for a longer time. The immature BBB enables the PLGA-PEG nanoparticles to more easily penetrate and enter the brain parenchyma in the early postnatal period and remain in the brain for a longer time. However, in the same-aged adult animals, there is almost no brain distribution, suggesting that the risk of enrichment of the nanozymes in the brain tissue of infants and young children cannot be ignored [74]. This may lead to different distribution volumes and target organ enrichment of nanozymes in tissues compared to adults; in the metabolism and excretion stage, in addition to the incomplete development of liver drug enzymes and glomerular filtration function, the unique development degree of the bile–intestinal circulation and the fluctuation of phagocytic activity of the reticuloendothelial system (such as liver, spleen, and bone marrow) in childhood may significantly alter the biological transformation pathways and clearance rates of nanozymes in the body. Currently, the metabolic kinetic data extrapolated from adult or animal models cannot accurately reflect the in vivo fate of nanozymes in different subgroups such as premature infants, infants, and school-age children. This critical data deficiency not only makes it difficult for us to predict the effective therapeutic concentration window of nanozymes in children’s bodies, but also prevents the assessment of potential accumulation risks and clearance half-life in multiple organs, thereby seriously hindering the formulation of reasonable drug administration schemes (such as drug dosage, administration interval, and treatment duration design) that comply with pediatric ethical requirements. In the future, the use of microsampling techniques, physiological pharmacokinetic modeling, and pediatric-specific animal models should be prioritized to fill this critical data gap.

#### 5.1.3. Developmental Toxicology

Developmental toxicology focuses on the health effects that result from the exposure of exogenous substances during specific developmental stages and that can persist into adulthood and even across generations. Due to its unique physical and chemical properties (small size, high surface area, catalytic production of reactive oxygen/free radicals), nanozymes may exhibit different toxic characteristics from those of adults during the critical periods of children’s development. Neurodevelopmental toxicity is the primary concern. As mentioned earlier, the blood–brain barrier (BBB) undergoes a gradual maturation process from the embryonic stage to the infancy stage. During this period, if nanozymes pass through the immature BBB and enter the brain parenchyma, they may affect neuronal migration, axonal guidance, and synaptic plasticity by inducing oxidative stress, activating microglia and myelin formation, etc. Epidemiological evidence indicates that exposure to ultrafine particulate matter in the air during the early stages of life is associated with an increased risk of autism spectrum disorders and attention deficit hyperactivity disorder. This provides a warning signal for the neurodevelopmental toxicity of nanozymes. Immune developmental toxicity should also not be overlooked. Perinatal exposure to aluminum oxide nanoparticles can pass through the placenta from the mother to the fetus and accumulate in the hippocampus of the offspring, causing oxidative stress and impairments in learning and memory. This suggests that there is a transgenerational neurodevelopmental toxicity risk associated with nanoparticle exposure during pregnancy and lactation [75]. The period from the fetal stage to 2 years after birth is a critical window for the development and establishment of tolerance in the immune system. If nanozymes are extensively taken up by antigen-presenting cells during this stage, they may interfere with the negative selection of T cells in the thymus and the differentiation of regulatory T cells by altering the maturation state and cytokine secretion pattern of dendritic cells. This can ultimately disrupt the establishment of immune tolerance. After entering the body, nanozymes adsorb plasma proteins on their surface to form a protein corona. This not only can be recognized and rapidly cleared by the mononuclear phagocytic system, shortening the circulation half-life, but also may mask the catalytic active sites, resulting in a decrease in the expected anti-inflammatory effect. Secondly, metal-based nanozymes with higher surface charge can directly activate the alternative pathway, release allergic toxins, and induce non-IgE-mediated activation-related pseudo-allergic reactions, which pose a greater risk in children requiring repeated administration. Moreover, some components of nanozymes can promote the release of pro-inflammatory factors by activating the TLR4/NF-κB pathway or the NLRP3 inflammatory body, resulting in an inflammatory amplification effect contrary to their anti-inflammatory purpose. More importantly, the immune system is in a critical window for establishing tolerance and shaping balance. The continuous low-level immune activation or interference caused by nanozymes may disrupt the Th1/Th2 balance, affect the differentiation of regulatory T cells, and theoretically increase the risk of long-term autoimmune or allergic diseases. In terms of reproductive and developmental toxicity, the accumulation of nanozymes in the gonads (ovaries and testes) may interfere with meiotic division of germ cells, synthesis of sex hormones, or support cell functions. It is particularly noteworthy that some nanozymes containing metal elements (such as iron-based and cerium-based) may generate ·OH through a similar Fenton reaction, causing DNA damage and epigenetic modification changes in germ cells, and the effects may be transmitted to the next generation. The fundamental dilemma faced by the current toxicological evaluation system is that conventional acute toxicity experiments cannot capture the delayed effects during the developmental process; while traditional two-generation reproductive toxicity experiments can assess reproductive and developmental toxicity, they have not designed specific exposure windows and endpoint indicators for nanomaterials. Therefore, it is urgent to establish a life cycle toxicological evaluation framework for pediatric nanomedicines, precisely anchoring the exposure window to key developmental stages such as organ development period, perinatal period, and adolescence, and incorporating endpoint indicators such as neurobehavioral tests, immune function assessment, reproductive endocrine detection, and multi-generation genetic effect tracking to systematically reveal the developmental toxicity spectrum of nanozymes.

#### 5.1.4. Large-Scale Production and Quality Control

The clinical translation of pediatric nanomedicine faces dual regulatory thresholds: one is the general requirements as a nanomedicine, and the other is the special regulations for the pediatric population. The FDA requires pediatric research plans to be included in a new drug application under the Pediatric Research Equity Act and the Best Children’s Medicines Act unless an exemption or extension is granted. For nanomedicines, the FDA updated its Guidance for Industry: Drugs Containing Nanomaterials in 2022, emphasizing the need to provide data on physicochemical properties, biodistribution in vivo, and clearance of nanoparticles, and requiring special attention to immunotoxicity and developmental toxicity in non-clinical studies. In China, the State Food and Drug Administration further issued the Technical Guidelines for Clinical Pharmacology Research in Children in 2023, which clarified the special requirements for pharmacokinetic studies in pediatric population. However, there are still obvious blind spots in the current regulatory system: there are no joint guidelines specifically for “pediatric nanomedicines”, most of the existing guidelines are independent requirements for “nanomedicines” and “pediatric drugs”, and there is a lack of systematic provisions for the intersection areas of the two (such as the dynamic changes in the biodistribution of nanomaterials during children’s development, and the quality property standards for pediatric nanomedicines). Moreover, the ethical challenges faced by pediatric clinical trials (informed consent, principle of minimal risk, appropriateness of placebo control) are more prominent in the nanomedicine field, where the risk benefit assessment is further complicated by the uncertainty about the long-term in vivo fate of nanomaterials.

Firstly, the metabolic kinetic data of nanozymes in pediatric clinical applications is lacking, which is one of the most critical scientific bottlenecks currently restricting the transition of nanozymes from basic research to clinical application. This deficiency is not merely a simple lack of data; it involves a systematic gap throughout the entire process from absorption to excretion. To date, only a few studies have measured the PK parameters and liver and spleen accumulation characteristics of PLGA-PEG nanoparticles in neonatal rats, and most of the data on the biodistribution and clearance of the nanoenzyme system in preterm infants and children of all ages are still blank [74,76]. Specifically, children are not merely smaller versions of adults; their dynamic physiological development directly affects the in vivo behavior of nanozymes: during the neonatal and infantile periods, the pH value of the gastrointestinal tract is relatively high (close to neutral), pancreatic enzymes and bile acid secretion are insufficient, and the intestinal barrier function is not yet mature. These factors may all change the stability, aggregation state, and transmembrane transport efficiency of nanozymes after oral administration, making the bioavailability difficult to predict. Secondly, the blood volume of children accounts for a higher proportion of their body weight, and the plasma protein concentration is lower. Moreover, the functions of the blood–brain barrier and blood–pulmonary barrier gradually improve with the development stage. This means that the free concentration of nanozymes in the blood circulation, the distribution volume to tissues (especially key organs such as the brain and lungs), and the targeting enrichment ability all differ significantly from those of adults, potentially increasing the risk of non-specific accumulation. Furthermore, the activity of liver drug enzymes (such as the CYP450 family) in childhood shows a dynamic developmental curve from low to high and then gradually adjusting. The glomerular filtration rate and renal tubular secretion function of the kidneys also reach adult levels only after several months or even years after birth. As an exogenous particle, the capture and degradation efficiency of nanozymes by the reticuloendothelial system in the liver, spleen, etc., as well as the clearance rate through the kidneys or bile, will all undergo nonlinear changes with the increase in the child’s age.

Currently, extrapolating doses based on adult data or findings from acute toxicity tests does not accurately represent how nanozymes behave in specific groups like premature infants, infants, and school-aged children. This approach also fails to identify the effective therapeutic range and the limits for cumulative toxicity. The lack of essential data hinders preclinical studies from developing scientifically valid dosing guidelines regarding the amount, frequency, and duration of treatments. As a result, pediatricians struggle to make informed decisions about potential benefits and unknown risks. To address this gap, it is crucial to adopt methods such as microsampling techniques, physiological pharmacokinetic modeling, and animal studies that reflect different developmental stages. This approach is urgently needed for future research in this field.

Secondly, for complex nanozyme systems that carry drugs or modify targeting molecules, the establishment of quality standards is far from being a simple extension of traditional chemical drugs. Instead, it faces a full-chain, multi-dimensional technical challenge ranging from physical and chemical characterization to biological activity evaluation. Such nanozymes are no longer a single active component but a dynamic composite system composed of a nano-carrier, enzyme active center, drug loading, and targeting modification layer. Their structural integrity and functional synergy are highly susceptible to environmental factors. On one hand, there is a lack of simple, standardized detection methods for precisely determining the number of drug molecules loaded on individual nanoparticles, the density of targeting ligands, and the number of metal active sites of the mimetic enzyme, while ensuring high consistency within and between batches. The slight fluctuations in drug loading, encapsulation rate, and ligand coupling efficiency can lead to significant differences in in vivo efficacy and targeting. Secondly, unlike traditional formulations, the drug release of such systems not only depends on diffusion or matrix erosion but is also influenced by the changes in enzyme activity of the nanozymes in the lesion microenvironment (such as pH, reactive oxygen concentration). How to establish an in vitro release medium that can simulate the complex physiological and pathological conditions in the body during the developmental period of children and scientifically evaluate its “on-demand release” or “enzyme-controlled release” behavior is a current methodological challenge. After targeting molecule modification, can it maintain its homing ability in complex body fluids without being blocked by opsonins? Does the chemical modification process lead to the attenuation of the catalytic activity of the nanozymes? Currently, there is a lack of effective means to monitor the entire process of “targeting-endocytosis-drug release-enzymatic reaction” in vivo, making it difficult to establish truly reflective quality evaluation indicators for in vivo efficacy. Finally, after entering the body fluid environment of children, the complex nano surfaces are prone to adsorb proteins and lipids to form “biological caps”, which may not only mask the targeting molecules, change particle size and surface charge, but even potentially trigger immune recognition. The current physicochemical indicators (such as particle size distribution, Zeta potential) often fail to predict their actual state and fate in the body.

Therefore, for such complex formulations, traditional drug or common nanomaterial quality control standards cannot be simply applied. The transition from laboratory-scale synthesis of nanozymes to GMP production at the kilogram level involves not only an expansion in scale but also a series of systematic challenges related to process robustness, quality consistency, and aseptic assurance. Ensuring batch consistency is the most crucial requirement of GMP. For complex nanozymes systems, traditional quality indicators (particle size, potential, purity) are no longer sufficient to fully reflect the product performance. It is necessary to introduce the concept of “quality derived from design”, establish a design space that includes key material properties and key process parameters, and achieve real-time monitoring through process analytical technology. In the future, it is urgently necessary to develop a quality control system based on the mechanism of action, which incorporates multiple advanced characterization techniques (such as asymmetric field-flow fractionation, single-particle inductively coupled plasma mass spectrometry), and combines the validation of multiple batches of in vivo efficacy and kinetics. A comprehensive quality control system covering “structural integrity-catalytic activity-targeting function-drug release behavior” should be established to ensure the safety, efficacy, and quality controllability of pediatric drugs.

### 5.2. Prospects for Future Research Directions

#### 5.2.1. Development of Intelligent Responsive Nanozymes

The future research on nanozymes will go beyond the traditional passive targeting or single functional modification, and will evolve towards a higher level of “intelligence” and “environmental adaptability”. The future research on nanozymes will go beyond the traditional passive targeting or single functional modification, and will evolve towards a higher level of “intelligence” and “environmental adaptability”. Specifically, this intelligent response mechanism can be designed based on the unique microenvironmental characteristics of pediatric inflammatory diseases:

(1) Multidimensional pathological signal perception: The inflammatory lesions in children (such as the airways of asthmatic patients, the intestinal mucosa of inflammatory bowel disease patients, and the vascular endothelium during the secondary inflammatory storm after infection) often exhibit complex microenvironmental characteristics, including local acidosis (with pH values dropping to 6.0–6.5), abnormally elevated levels of ROS (such as excessive generation of H_2_O_2_ and •OH), and excessive expression of specific matrix metalloproteinases or myeloperoxidase. Intelligent nanozymes should be able to integrate the recognition of these combined signals, rather than relying solely on a single factor for triggering.

(2) Diversification of response activation patterns: This “inflammation-triggered” activation can be achieved through various molecular mechanisms. For instance, by using pH-sensitive polymers for coating or charge-flipping properties, the active center of the nanozyme can be blocked under normal physiological pH (7.4), and upon entering the acidic inflammatory microenvironment, the conformational change exposes the catalytic site; or the active components of the nanozyme can be anchored on the surface of the carrier through linkers that can be cleaved by ROS, and only when there is an excess of ROS will they be cleaved and released, thereby achieving the targeted activation of catalytic activity.

(3) The integration of treatment and monitoring: More advanced intelligent nanozymes can also integrate feedback regulation mechanisms. That is, after being activated by inflammatory signals and exerting antioxidant or immune regulatory effects, as the local microenvironment becomes more normalized (such as a decrease in ROS levels and a restoration of pH), they can automatically reduce their own activity or initiate clearance procedures to avoid excessive treatment. At the same time, responsive imaging probes can be introduced to enable them to generate detectable optical or magnetic resonance signals simultaneously when activated, allowing for real-time visual monitoring of the treatment process.

Achieving this “inflammation-triggered” precise activation will bring dual clinical benefits: On the one hand, it enables efficient and concentrated anti-inflammatory treatment at the lesion site, ensuring that the nanozyme functions to the maximum extent at the “correct location and at the correct time”; on the other hand, it keeps normal tissues such as the liver, spleen, and kidneys or non-lesion areas in a “silent” state, significantly reducing non-specific damage to healthy cells and potential chronic toxicity. Especially for children whose organ systems are not yet fully developed, this “on-demand treatment” strategy can maximize treatment specificity and significantly reduce systemic side effects caused by off-target effects, opening up a new path of precise treatment for pediatric inflammatory diseases that is both highly effective and safe.

#### 5.2.2. Innovative Design of Biodegradable Nanozymes

In response to the extremely high demands for biological safety from the special group of pediatric patients, the future research strategies for nanozymes are undergoing a profound transformation from “performance priority” to “equally emphasizing safety and functionality”. The core direction of this transformation is to shift the research focus from inorganic or noble metal-based materials that are difficult to degrade, to nanozyme systems constructed from essential elements of life and natural organic substances. This design innovation is mainly reflected in the following aspects:

(1) The new type of nanozymes tend to utilize the naturally occurring essential trace elements in the human body, such as iron, manganese, zinc, and selenium, as well as naturally active organic substances. These elements themselves are the cofactors or key components of various antioxidant enzymes in the human body (such as GPx containing selenium, SOD containing copper–zinc or manganese). Using them as building blocks means that after the nanozymes complete their anti-inflammatory mission, their degradation products are not “foreign substances”, but rather can directly enter the original iron metabolism pool, selenium utilization pathway, or amino acid cycle of the human body as nutrients.

(2) Biocompatible nanozymes can be designed with “degradable switches” incorporated during their construction. For instance, nanozymes based on ferritin or iron-containing nanoclusters can be decomposed into iron ions and peptides within the acidic environment of lysosomes and through specific enzymatic actions within cells. The latter can be reused by the cells through the transferrin pathway or excreted through the kidneys. Nanozymes based on selenium (such as selenocysteine derivatives) can participate in the redox cycle in the body. Their metabolites can be easily eliminated through urine or bile, avoiding long-term retention in the reticuloendothelial system such as the liver and spleen.

(3) For this special group of children, especially newborns and infants whose organ systems are in a period of rapid development, any foreign materials that are difficult to be eliminated may pose a potential developmental toxicity threat. By constructing nanozymes using essential elements for life, it is possible to fundamentally avoid the accumulation risk of inorganic materials in the developing nervous system and reproductive system. Even if the clearance rate is slower due to the physiological characteristics of children (such as a lower glomerular filtration rate), these degradation products will not form permanent tissue deposits like non-degradable materials, thereby significantly reducing the concerns about chronic toxicity.

(4) These nanozymes composed of essential elements not only exert anti-inflammatory effects but also their degradation products may bring additional health benefits. For instance, in children with inflammatory bowel disease, zinc-based nanozymes not only eliminate excessive ROS but also the zinc ions released after degradation can promote intestinal mucosal repair and immune function regulation; selenium-based nanozymes, after exerting antioxidant effects, can supplement this important immune nutrient for children in selenium-deficient areas, achieving the integration of treatment and nutritional support.

(5) Unlike inorganic nanozymes that require stringent conditions for synthesis, biogenic nanozymes can often simulate the catalytic mechanism of natural enzymes under mild physiological conditions. They bind to substrates through weak interactions such as coordination bonds and hydrogen bonds, exhibit high catalytic efficiency, and are conducted under mild reaction conditions, which better conform to the physiological characteristics of the microenvironment in children’s bodies and reduce local tissue stimulation caused by excessive catalysis or non-specific reactions.

Therefore, shifting the research focus to essential elements of life and natural organic substances is not only a change in material selection, but also a leap in the design philosophy of nanozymes—evolving from “external therapeutic tools” to “temporary functional components that can be accepted by the body and participate in metabolism”. This “innocuous in the body and completely disappears after the disease is cured” design concept is expected to fundamentally solve the long-standing safety problems in the pediatric clinical transformation of nanozymes, providing truly safe and controllable precise treatment options for children with inflammatory diseases.

#### 5.2.3. Specialized Delivery System and Formulation Optimization for Children

The active development of friendly dosage forms suitable for children is the key bridge that enables nanozymes to move from the laboratory to pediatric clinical practice. Children are not miniature versions of adults; their acceptance of the dosage form, swallowing ability, taste preferences, and psychological feelings are all closely related to their developmental stage. Therefore, the formulation development of nanozymes should break away from the traditional framework of tablets and capsules and shift towards developing more diverse dosage forms that better suit the physiological and psychological characteristics of children. Specifically, a systematic layout can be carried out from the following dimensions:

(1) Development of multi-pathway dosage forms: To address the difficulty of infants swallowing tablets, a nanozyme oral suspension with good taste, uniform dispersion, and high physical stability was developed. The unpleasant odors of metals or drugs were masked through flavoring techniques, and sweeteners and fragrances were used to enhance children’s acceptance, ensuring effective release at the intestinal lesion sites (such as inflammatory bowel disease). For respiratory system diseases such as childhood asthma and post-infection airway inflammation, the nanozyme was prepared into inhalable dry powder or aerosol inhalation liquid with an appropriate aerodynamic particle size (1–5 μm). This dosage form bypasses the first-pass effect of the liver and directly delivers the drug to the airway and alveolar lesions, with rapid onset and low systemic exposure. When designing, the differences in airway diameters and inhalation flow rates among children of different ages need to be considered, and the face mask adaptability of the inhalation device should be optimized. For superficial inflammatory conditions such as pediatric AD and eczema, nanozyme transdermal patches or hydrogels were developed. Taking advantage of the small particle size of nanozymes, they can penetrate the damaged skin barrier to achieve local sustained release. At the same time, the patches can be designed with cartoon patterns, and the gel can have a fresh fragrance to reduce the resistance of the children. For children who cannot take medicine orally or have severe vomiting (such as high fever convulsions, severe infections), nanozyme rectal suppositories or foams were developed to provide safe and convenient alternative drug delivery routes.

(2) Delivery strategies for crossing developmental physiological barriers: There are significant differences in the physiological barrier functions of children at different age stages. The delivery system needs to be designed accordingly. For example: Newborns have less gastric acid secretion and a higher gastric pH value, and the intestinal mucus layer is thinner and more permeable. When designing oral nanozymes, stability in a higher pH environment needs to be considered, and mucosal penetration modification (such as PEGylation) or mucosal adhesion modification should be utilized to regulate their retention time and absorption degree in the intestine. For infants with weaker ciliary clearance function in the airway and different composition of pulmonary surfactants compared to adults, inhalation preparations need to be designed as nanozymes that can escape macrophage phagocytosis and effectively penetrate the airway mucus layer, while ensuring their catalytic activity remains intact in the presence of pulmonary surfactant. Newborns and infants have thin keratin layers and high water content, with higher percutaneous permeability than adults. The percutaneous delivery system needs to take advantage of this feature to achieve efficient transdermal delivery, but must also strictly control the transdermal rate to prevent excessive systemic absorption due to incomplete skin barrier function. This can be achieved through technologies such as microneedle patches. When treating pediatric central nervous system inflammation (such as purulent meningitis), the window period when the blood–brain barrier permeability is relatively high during development, or through endogenous transport systems mediated by transferrin receptors, such as transcytosis, should be utilized to achieve targeted delivery of nanozymes to the brain parenchyma. In addition to the formulation itself, dedicated pediatric-specific delivery devices (such as quantitative spray pumps, interesting inhalers, and clearly marked droppers) need to be developed, and single-dose packaging should be adopted to ensure precise administration. In the early stages of formulation development, taste and appearance preference tests for pediatric populations should be introduced to make nanozyme drugs truly “child-friendly” products.

Through the deep integration of the above formulation innovations and delivery strategies, not only can nanozymes accurately reach the lesion sites in children of different ages and exert their therapeutic effects, but also it can fundamentally solve the problems of treatment interruption or inaccurate dosage caused by the difficulty in drug administration for pediatric patients, thus paving the way for the wide application of nanozymes in pediatric inflammatory diseases.

#### 5.2.4. Construction of Regulatory Science and Clinical Transformation Pathways

Promoting the clinical transformation and application of nanozymes in the pediatric field cannot be accomplished independently by a single discipline. Essentially, it is a system engineering project that requires in-depth collaboration among multiple disciplines throughout the entire research and development process. Future development must break through disciplinary barriers and encourage materials scientists, pediatricians, toxicologists, and drug regulatory agencies to engage in early involvement and regular in-depth cooperation from the project conception stage, jointly establishing a new paradigm for pediatric nano-drug research that is both in line with scientific logic and adheres to ethical boundaries, as well as being forward-looking and practical. Specifically, this collaborative innovation and prudent advancement strategy can be carried out at the following levels:

(1) The traditional toxicological evaluation system is mainly based on adult animal models and adult medication experience, and it is difficult to directly apply to children. Therefore, it is urgent for materials scientists to reveal the physical and chemical property changes in nanozymes in the simulated environment of children’s development, for pediatricians to provide physiological and pathological characteristics and clinical needs at different developmental stages, for toxicologists to design development toxicity research plans covering pregnant, lactating, and adolescent animals, and to jointly discuss with drug regulatory agencies to establish a set of non-clinical safety evaluation guidelines for pediatric nanomedicines. This guideline should focus on the specific toxic endpoints unique to children, such as immune system development interference, neurobehavioral effects, reproductive system damage and endocrine interference, and clearly define what degree of data accumulation can exempt some long-term experiments, so as to accelerate the entry of urgently needed varieties into clinical trials while ensuring safety.

(2) As a vulnerable group, children face higher ethical thresholds in clinical research. It is necessary for pediatricians, ethicists, patient representatives, and regulatory agencies to jointly establish an ethical review framework for innovative nanozyme therapies. This framework should clearly define under what risk–benefit ratio pediatric initial human trials can be conducted, how to obtain informed consent from children of different ages (such as assent from young children and consent from parents), and how to establish an independent data safety monitoring committee during the research process to ensure that the rights and interests of the participating children are protected to the greatest extent.

(3) Considering the high risks and uncertainties associated with the development of pediatric nanomedicines, it is not advisable to pursue a broad coverage of extensive indications. Instead, a “focus on key areas and gradual breakthroughs” strategy should be adopted. Priority should be given to selecting those rare and severe pediatric inflammatory diseases that currently have “no effective treatment” or for which existing therapies (such as long-term high-dose hormones and immunosuppressants) carry extremely high risks and even lead to disability or death as the breakthrough points for clinical translation. For example, severe refractory juvenile idiopathic arthritis children who are resistant to traditional disease-modifying antirheumatic drugs and biologics. Early-onset inflammatory bowel disease, especially those who develop the disease in infancy, have poor response to existing treatment regimens, and severely affect growth and development. Post-infection cytokine storm syndrome: such as severe COVID-19 infection in children or hemophagocytic syndrome, with a dangerous course and an urgent need for rapid and precise anti-inflammatory intervention. Genetic autoinflammatory diseases, such as Cryopyrin-related periodic syndrome, which is caused by abnormal activation of the innate immune system, and the antioxidant and immunomodulatory properties of nanozymes may directly target the pathological core.

(4) In the field of rare diseases, conducting traditional randomized controlled trials often encounters practical challenges such as insufficient case numbers and ethical restrictions. It is advisable to consider, under strict supervision and ethical review, expanding compassionate use and real-world research to gradually accumulate data on the efficacy and safety of nanozymes in specific pediatric subgroups. These clinical data not only provide a basis for subsequent indication expansion but can also provide reverse guidance for materials scientists to optimize the configuration and dosage form of nanozymes, forming a virtuous cycle of “clinical feedback-laboratory optimization-re-clinical validation”.

(5) Encourage the establishment of a pediatric nanomedicine collaborative innovation center that is guided by the government, led by academic institutions, involving enterprises, and with the participation of regulatory agencies. In the early stages of the project, through the “scientific advice” channel of the regulatory agencies, pre-communications should be conducted on key issues such as the non-clinical research design of the product, pediatric clinical development plans, and quality controllability standards, to avoid waste of research resources due to research design flaws and to increase the success rate of transformation.

Through this multi-disciplinary integration and the prudent and practical approach of implementation, we can gradually uncover the therapeutic potential of nanozymes in pediatric inflammatory diseases while ensuring the maximum safety of the children as subjects. The first-hand clinical experience accumulated from the most urgent rare severe disease areas will serve as the scientific foundation for building a broader pediatric application basis. Eventually, this innovative technology will benefit more children suffering from inflammatory diseases and their families.

## 6. Summary and Outlook

The current diagnosis and treatment of pediatric inflammatory diseases call for innovative strategies that can balance efficiency, safety, and developmental specificity. This review systematically explores the significant potential and application prospects of nanozymes—an interdisciplinary frontier that combines nanotechnology with enzymatic catalysis—in addressing this challenge. Through meticulous physical and chemical design, we can endow nanozymes with the powerful ability to mimic key antioxidant enzymes such as SOD, CAT, and GPx, and further achieve their precise delivery to inflammatory lesions through surface functionalization. More importantly, the role of nanozymes is not merely that of passive antioxidants, as they can actively intervene in the core processes of inflammation, by eliminating ROS, reprogramming macrophage polarization, and inhibiting NLRP3 inflammasome activation, among other mechanisms, fundamentally reshaping the inflammatory microenvironment. This provides a new treatment paradigm beyond traditional drugs for typical pediatric severe inflammatory diseases such as NEC, JIA, asthma, and IBD. Moreover, its outstanding performance as a multifunctional platform in integrated diagnosis and treatment and collaborative therapy has further elevated the precision and efficacy of disease management to a new level.

However, the path towards pediatric clinical application still has a long way to go. The long-term biological safety of nanozymes, precise individualized dosing strategies, and quality control for large-scale production are the hurdles that must be overcome for their successful transformation. Looking to the future, the research focus should shift to developing intelligent-responsive and biodegradable next-generation nanozymes, and conducting in-depth systematic toxicology and pharmacokinetic studies targeting the pediatric population. In conclusion, emerging nanomedicine technologies represented by nanozymes are leading the paradigm shift in the diagnosis and treatment of pediatric inflammatory diseases with their unique charm. Although challenges remain on the road ahead, through close interdisciplinary cooperation and continuous exploration centered on children’s health, we have reason to believe that nanozymes will eventually evolve from a promising laboratory technology into a solid force safeguarding children’s lives and health and illuminating their growth path.

## Figures and Tables

**Table 1:** The mechanism and advantages of nanozymes in inflammatory diseases.

Nanozyme	Disease Type	Mechanism of Action	Experimental Model	Translational Stage	Advantages and Limitations	Refs.
BSA/HSA-MnO_2_	Breast cancer	CAT-like; pH/H_2_O_2_ responsive dissociation	4T1 Cell and tumor animal experiments	Preclinical in vivo	1. pH/H_2_O_2_ dual-response intelligent dissociation; 2. Enhance the EPR effect; 3. Not involving spontaneous tumor or metastasis models.	[10]
Au@Hollow MnO_2_ Nanomotors	Solid tumor	Fenton-like; glutathione depletion; CDT	2D cells and 3D Tumor multicellular spheres	Preclinical-principle verification	1. Biodegradable; 2. Good biocompatibility; 3. Double amplification CDT effect.	[12]
Prussian Blue	RA	SOD-like CAT-like	Osteoclast Cell and AIA animal experiments	Preclinical in vivo	1. Mimic activity of multiple antioxidant enzymes; 2. Good safety profile; 3. Long-term accumulation toxicity.	[13]
Fe–N_4_ SANzyme	Retinal vasculopathies	CAT-like	HRMECs cell and OIR animal experiments	Preclinical in vivo	1. Higher affinity; 2. Good biocompatibility; 3. Enhanced CAT-like activity.	[15]
MANZs	RA	SOD-like CAT-like Targeted delivery Immune reprogramming	Osteoclast Cell and CIA animal experiments	Preclinical in vivo	1. Good biocompatibility; 2. Improve retention time; 3. Biodegradable; 4. No clinical translational studies.	[17]
PAA-capped CeO_2_	Respiratory Syncytial Virus	SOD-like CAT-like	BEAS-2B cell and RSV animal experiments	Preclinical in vivo	1. Stable dispersion; 2. Good biocompatibility; 3. No clinical translational studies.	[19]
RBCm/FA-TA-CeO_2−x_	UC	SOD-like CAT-like GPx-like	RAW264.7 macrophages and UC animal experiments	Preclinical in vivo	1. Diverse enzyme activities; 2. Good safety profile; 3. No clinical translational studies.	[20]
Fe–N_4_ MOF	Diabetes Mellitus–induced Erectile Dysfunction	SOD-like CAT-like GPx-like	Cell and DMED model animal experiments	Preclinical in vivo	1.Diverse enzyme activities; 2.Single atom dispersion activity enhancement; 3.No clinical translational studies.	[21]
Ru-SAs/NCs@g-C_3_N_4_	OA	SOD-like CAT-like GPx-like	Cartilage cells and OA animal experiments	Preclinical in vivo	1. Enhancement of enzymes activity; 2. Good biocompatibility; 3. Long-term accumulation toxicity.	[22]
Mn@CeO_2_@Alg/Eud/Bn	UC	SOD-like CAT-like GPx-like	Cell and UC, TNBS colitis model animal experiments	Preclinical in vivo	1. Diverse enzyme activities; 2. Good safety profile; 3. No clinical translational studies.	[23]
PVP-capped Prussian Blue	UC	SOD-like CAT-like	RAW264.7 macrophages and UC animal experiments	Preclinical in vivo	1. Higher affinity; 2. Good biocompatibility; 3. Long-term accumulation toxicity.	[24]
RuCo-NSs	UC	SOD-like CAT-like GPx-like	Cell and UC, TNBS colitis model animal experiments	Preclinical in vivo	1. Diverse enzyme activities; 2. large surface area; 3. High exposure rate of active sites.	[25]
PAA–CeO_2_	Bacterial skin infection	Oxidase-like; Fenton-like CDT effect; GOx-like	MRSA and MRSA infection wound model animal experiments	Preclinical in vivo	1. self-sufficiency of H_2_O_2_; 2. Good biocompatibility; 3. No clinical translational studies.	[26]
N-BiOI NSs	Bacterial skin infection	Photothermal effect; POD-like	MRSA and MRSA infection wound model animal experiments	Preclinical in vivo	1. Triple synergy effect; 2. High-efficiency photothermal conversion; 3. No clinical translational studies.	[27]
PDA@CeO_2_ NPs	ALI	SOD-like CAT-like ·OH scavenging Photothermal effect	RAW264.7 macrophages and ALI animal experiments	Preclinical in vivo	1. Good biocompatibility; 2. Good safety profile; 3. No clinical translational studies.	[28]
Dex@MnO_2_–BSA	ALI	CAT-like; H_2_O_2_ responsive; Synergistic anti-inflammatory	RAW264.7 macrophages and ALI animal experiments	Preclinical in vivo	1. Good biocompatibility; 2. cycling stability; 3. No clinical translational studies.	[29]
Dex@MnO_2_–BSA	ASD	H_2_O_2_ responsive; CAT-like Synergistic anti-inflammatory	RAW264.7 macrophages and ALI animal experiments	Preclinical in vivo	1. Good biocompatibility; 2. cycling stability; 3. No clinical translational studies.	[30]
Lam–Pt NPs	ICH	SOD-like CAT-like Cellular reprogramming	BV2 cell and ICH model animal experiments	Preclinical in vivo	1. Excellent BBB; 2. Good safety profile; 3. No clinical translational studies.	[31]
PDA-MNs	AD	SOD-like CAT-like Photothermal effect; Immunoregulation	HaCaT cells and AD model animal experiments	Preclinical in vivo	1. Low metal toxicity; 2. Good biocompatibility; 3. No clinical translational studies.	[32]
CeO_2_–Exos	CIA	SOD-like CAT-like Immunoregulation	RAW264.7 macrophages and CIA animal experiments	Preclinical in vivo	1. Higher affinity; 2. Good biocompatibility; 3. No clinical translational studies.	[33]
Mn_3_O_4_ NPs	PD	SOD-like CAT-like GPx-like	SH-SY5Y cell and Subacute PD model animal experiments	Preclinical in vivo	1. Low cost; 2. Good biocompatibility; 3. No clinical translational studies.	[34]
CeO_2_–FD–Gel	UC	SOD-like CAT-like Bile acid metabolism	HT-29/HCoEpiC intestinal epithelial cells and UC model animal experiments	Preclinical in vivo	1. Good biocompatibility; 2. Good safety profile; 3. No clinical translational studies.	[35]

## Data Availability

No new data were created or analyzed in this study. Data sharing is not applicable to this article.

## Abbreviations

Neonatal necrotizing enterocolitis	NEC
Juvenile idiopathic arthritis	JIA
Inflammatory bowel disease	IBD
Collagen-induced arthritis	CIA
Atopic dermatitis	AD
Autism spectrum disorder	ASD
Ulcerative colitis	UC
Acute lung injury	ALI
Intracerebral hemorrhage	ICH
Crohn’s disease	CD
Osteoarthritis	OA
Rheumatoid arthritis	RA
Blood–brain barrier	BBB
Reactive oxygen species	ROS
Peroxidase-like enzyme	POD
Oxidase-like enzyme	OXD
Superoxide dismutase-like enzyme	SOD
Catalase-like enzyme	CAT
Glutathione peroxidase	GPx
Hydrogen peroxide	H_2_O_2_
Superoxide anion	·O_2_^−^
Hydroxyl radical	·OH
Polyethylene glycol	PEG
Magnetic resonance imaging	MRI
Photoacoustic imaging	PA
Aspartate proteolytic enzyme-3	Caspase-3
Triphenylphosphine	TPP
